# The Influence of Starting Plant Material on Ni@C-Type Composites’ Characteristics

**DOI:** 10.3390/ma18163784

**Published:** 2025-08-12

**Authors:** Kamil Dudek, Stanisław Małecki, Kamil Kornaus, Piotr Żabiński

**Affiliations:** 1 Faculty of Non-Ferrous Metals, AGH University of Krakow Mickiewicz Avenue 30, 30-059 Cracow, Poland; stanmal@agh.edu.pl; 2 Faculty of Material Engineering and Ceramics, AGH University of Krakow, Mickiewicz Avenue 30, 30-059 Cracow, Poland; kornaus@agh.edu.pl

**Keywords:** activated carbon, biomass, nickel, composite, adsorption

## Abstract

This study describes the development and characterization of materials based on activated carbon (AC). Pellets composed of dried biomass of willow, knotweed, and maple were formed and pyrolyzed to obtain different types of AC. Nickel (Ni) nanoparticles were synthesized on these materials using a bottom-up strategy by impregnating the carbons with a nickel nitrate solution. To characterize the surface and structure of these materials, SEM, MP-AES, and DSC-TGA techniques were employed. The ash content was analyzed to determine the input of mineral components in the carbons. The DSC-TGA results showed good thermal stability for each of the carbons, even at a temperature of 800 °C. BET analysis was also conducted, and the isotherms revealed well-developed surfaces for most of the specimens. The high efficiency of the impregnation process was confirmed by the MP-AES results: 165 mg of Ni was deposited on 1 g of carbon derived from maple leaves. The adsorbed Ni was well distributed across the carbon surfaces, as demonstrated in micrographs taken with the SEM-EDS apparatus. A comparison with similar materials reported in other studies was also performed.

## 1. Introduction

Nowadays, scientists are searching for new renewable sources of multifunctional materials with unique properties. A prime example of such a material is “Activated carbon (AC), also called activated charcoal, […] basically defined as black carbonaceous porous solid material with high specific surface area, reasonable pore size distribution, and high-degree of surface reactivity.” [1]. It is produced from carbonaceous sources such as biomass, petroleum pitch, lignite, or coal. The primary use of AC is filtering contaminants from water and air. It can be used in the form of powder, pellets, beads, or parisons. The size and quantity of AC’s pores depend on its source and production methods.

Preparation of activated carbon can be conducted with the use of physical or chemical methods. Prior to activation, a process called carbonization is sometimes performed.

Carbonization is a form of thermal treatment regarding a raw material, and its main purpose is to decrease the level of volatile organic compounds in the feedstock and increase its carbon content. Carbonization is mostly conducted via pyrolysis, in the temperature range of 300–900 °C, in an inert atmosphere (e.g., nitrogen or argon). A result of pyrolysis is a char with primary porosity. Such material contains large quantities of fixed carbon [2,3].

Physical activation relies on the partial gasification of a subsequently carbonized precursor or a raw lignocellulosic material in the presence of an oxidizing gas (mainly oxygen, carbon dioxide, or water steam, and sometimes a mixture of those gases). It is conducted in temperatures ranging from 800 to 1200 °C [4,5,6]. The benefit of such activation is that it does not require the use of corrosive chemicals, but the disadvantage of the process is the necessity of using a high temperature and long-time processing.

Chemical activation involves impregnating the feedstock or a subsequently carbonized raw material with an acid (e.g., phosphoric, sulfuric, or nitric acid), a strong base (e.g., sodium or potassium hydroxide), or a salt solution (e.g., calcium chloride or potassium carbonate). Next, the impregnated material is subjected to temperatures ranging from 400 to 1000 °C [7,8]. To achieve higher internal porosity, a strictly controlled stream of gas such as air, nitrogen, or argon can also be applied during heating. After the heat treatment, a carbon is washed to leach out the activating agent. In comparison with physical activation, the advantage of chemical activation is lower processing temperature and better porosity control, but its main flaw is the necessity of washing the samples after activation. It also requires the use of highly corrosive chemicals.

After activation, the resulting carbon is sieved or granulated to achieve a homogeneous consistency.

Due to their strong porosity, most ACs have a highly developed surface area. The surface area of commercially used ACs typically ranges from 500 to 3000 m^2^ per gram of material. ACs are capable of adsorbing small molecules, primarily through van der Waals forces or London dispersion forces. The iodine number is a key parameter used to characterize the adsorption capacity of activated carbon. Commercially available activated carbons usually have an iodine number ranging from 500 to 1200 mg/g.

A particularly interesting group of ACs is obtained from biomass. Aside from water and air purification, these ACs have a much wider range of applications and are produced from various raw materials, such as lignite, coir, wood, willow peat, coconut husk, bamboo, and cellulose [7,8,9,10,11,12,13,14]. Each of these feedstocks has a unique structure that influences the properties of the resulting carbon, determining its application.

For instance, if AC is intended to be used as a catalyst support, it should possess a high specific surface area (SSA) to offer numerous easily accessible active sites. It should also have low sulfur content to prevent rapid catalyst deactivation. When used as an adsorbent, AC must exhibit not only a high SSA but also a high iodine number, which indicates its adsorption capacity. This parameter is particularly crucial for ACs intended for use in air or water filtration. Conversely, if AC is to serve as a storage for gases such as methane or hydrogen, it should have a high volume of micropores.

ACs have been successfully applied across various fields. For example, coconut shell-derived ACs can adsorb harmful gases such as CO_2_, CO, and NO_x_ [15], thereby improving air quality. This functionality is attributed to their exceptionally large surface area and high microporosity, which provide abundant sites for gas physisorption via van der Waals interactions. Their moderate surface energy variability offers both selectivity and reusability, making them easy to regenerate.

Similarly, ACs produced mechanochemically from chestnut tannin demonstrate excellent CO_2_ capture capabilities [16]. These carbons possess a highly complex microporous structure, offering size-matched adsorption sites for effective physisorption. A two-step activation process—potassium citrate treatment (to enhance pore volume) followed by CO_2_ activation at 700 °C (to increase surface area)—enables both chemisorption and physisorption, resulting in high uptake even under ambient conditions. Naturally occurring nitrogen in the precursor acts as Lewis basic sites that attract acidic CO_2_ molecules.

Wood-derived ACs are suitable for adsorption-based cooling systems [17] thanks to their high BET surface area and pore volume. These properties allow them to adsorb refrigerants in large quantities (over 1 kg of refrigerant per 1 kg of adsorbent). Their physisorption mechanism, with modest heat release, ensures efficient adsorption/desorption cycles, even at moderate temperatures (~30–50 °C).

Rice husk-derived carbon effectively removes Fe(III) and Mn(II) ions from aqueous solutions [18]. It has a well-developed surface area, excellent porosity, and a high density of surface functional groups, enabling strong metal ion binding through ion exchange and surface complexation. These properties arise from both the porous nature of the rice husk and the chemical activation with H_3_PO_4_, which enhances porosity and introduces functional groups such as –OH and –COOH.

Carbon derived from palm biowaste efficiently removes heavy metals (e.g., Pb(II)) through electrostatic attraction between negatively charged surface groups (like –COOH) and positively charged metal ions. It also utilizes mechanisms such as complexation with oxygen-containing groups, ion exchange, and surface precipitation at higher pH levels [19]. It is also effective in dye adsorption, such as for methylene blue [20], via π–π interactions with graphitic domains, hydrogen bonding with functional groups, and electrostatic attraction to the negatively charged surface.

Cabbage-based biochar exhibits good adsorption capacity for dyes and antibiotics [21]. Antibiotic molecules are adsorbed through complexation with Mg^2+^ ions on the carbon surface, hydrophobic interactions, and π–π stacking with aromatic structures in the biochar matrix.

For hydrogen sulfide removal, hydrothermally synthesized ACs from spent coffee grounds, aloe vera, and corncob have shown promising results [22]. These carbons are doped with metal oxides (ZnO, CuO, and Fe_2_O_3_), which react with hydrogen sulfide to form insoluble precipitates such as CuS. Their high porosity enhances gas diffusion and interaction with active sites.

In catalysis, AC serves as a versatile support for various catalysts. For example, sugarcane bagasse-derived AC supports Co nanoparticles used in hydrogen peroxide-sensing systems [23]. Cobalt acts as a redox-active center, facilitating electron transfer, O–O bond cleavage, and proton-coupled electron transfer in H_2_O_2_.

For the hydrogen evolution reaction (HER), catalysts supported on ACs composed of sugarcane bagasse, catkin, and other biomass sources have been developed using Mo as the active component [24]. In addition to pyrolysis, synthesis involves hydrothermal doping with Co, Mo, and N, along with ultrasonication. Biomass-derived carbon functions as both an electronic and structural modulator, improving the Volmer–Heyrovsky HER pathway by lowering kinetic barriers and increasing active site availability. Another HER catalyst is derived from pyrolyzed cashew nut skin [25].

Phosphorus-doped AC from starch, synthesized via hydrothermal treatment followed by carbonization, is an effective catalyst for n-heptane dehydroaromatization [26]. This catalyst activates C–H bonds in n-heptane, producing aromatic compounds. The acidic sites introduced by phosphorus doping enhance catalytic activity.

Wu et al. developed an oxygen reduction reaction (ORR) catalyst using a two-step synthesis involving chemical activation and a constant-pressure-drop funnel technique [27]. This method ensures uniform metal dispersion on the AC surface. The nitrogen-rich matrix prevents Co agglomeration, increasing active site density. Another ORR catalyst was prepared by thermochemically activating wood with alkali and phosphoric acid, followed by nitrogen doping [28]. Adjusting surface chemistry via different activators modulates electron density around N-sites, improving ORR kinetics. A Pt/C catalyst supported on steam-activated sawdust also performs well in ORR applications [29]. Pyrolyzed wood chips activated with NaOH and doped with Mn and N form another ORR catalyst [30]. All four systems mentioned above support the direct four-electron reduction of oxygen to water, minimizing peroxide intermediate formation and enhancing reaction rates.

Alkaline activation is also part of the synthesis of a hydrochar-supported catalyst for converting motor oil into diesel-range hydrocarbons [31]. This catalyst promotes C–C bond cleavage at high temperatures by stabilizing radicals through surface functional groups and facilitating proton transfer or H-abstraction, yielding C_10_–C_20_ hydrocarbons instead of gas or coke.

To produce butyl esters from bio-oil, Ibrahim et al. synthesized a bimetallic catalyst supported on rice straw-based AC [32]. This catalyst promotes esterification between organic acids and 1-butanol. The process is catalyzed via Lewis–Brønsted acid duality, with Lewis sites activating the carboxyl group and Brønsted sites facilitating proton transfer and nucleophilic attack.

All the examples from the literature stated above prove the versatility and utility of biomass-based activated carbons. They inspired the authors of this study to develop a series of similar materials based on biomass sourced from different plant species. The goal of this work was to synthesize a set of new materials based on renewable sources, characterize them, and find their most suitable application. For this study, different plants and plant residues were selected. There are reports of obtaining ACs based on willow catkins [33] and giant knotweed [34]. However, there are no articles about such materials based on maple leaves. Additionally, there is no information about composites of Ni and willow leaf-based carbon or Japanese knotweed-based carbon. In conclusion, the materials described in this work are new. The precursors were pyrolyzed under the same optimal conditions established in the previous study [35].

## 2. Materials and Methods

### 2.1. Materials’ Preparation

Activated carbon preparation: Knotweed and willow shoots, as well as fallen maple leaves, were collected from the suburban area of Jaworzno (Poland, Silesian Voivodeship) at the beginning of November 2023. The leaves were then separated from the stems and dried for one month in a warm environment. Afterwards, the leaves were crushed by hand and ground into a powder using a mincer (ML—maple leaves, KL—knotweed leaves, and WL—willow leaves). The resulting powder was placed into a 13 mm diameter mold and pressed into pellets using a hand-operated hydraulic press under a pressure of 10 MPa. The pellets were pyrolyzed in a tube furnace (Czylok, Jastrzębie-Zdrój, Poland) under a nitrogen atmosphere (99.999% purity, Air Liquide, Kraków, Poland) with a flow rate of 10 mL/min at 700 °C for 1 h. The heating rate was 10 °C/min. The obtained carbons’ yields are presented in Table 1.

Ni/AC composite preparation: 18.45 g of Ni(NO_3_)_2_·6H_2_O (WARCHEM, Zakręt, Poland) was dissolved in 1 L of distilled water to prepare a 63 mM nickel solution. Then, 3 g of ML, KL, and WL carbon pellets were placed into separate glass bottles, each containing 200 mL of the prepared nickel solution. The bottles were sealed with screw caps and sonicated for 15 min. Next, the bottles were placed in a heated water bath with a shaker (ELPIN, Lubawa, Poland), set to 80 °C. The samples were heated and shaken for 2 h, after which the carbon pellets were removed from the bottles. The filtrate was collected and stored, while the impregnated carbon pellets were transferred to beakers and dried at 105 °C for 2 days. After drying, the impregnated charcoals were calcined in a tube furnace. The temperature was increased from 23 °C at a rate of 10 °C/min to 500 °C and held at that temperature for 3 h under a nitrogen stream (10 mL/min). All samples were collected, stored in zip-lock bags, and analyzed to determine the relationship between the preparation conditions and their physicochemical properties.

### 2.2. Characterization Methods

BET Analysis: The isotherms were obtained using a Micromeritics ASAP 2010 (Micromeritics Instrument Corporation, Norcross, GA, USA) apparatus. The analysis cell was maintained at a temperature of −195.8 °C. Samples were degassed at 350 °C for 24 h before measurement. The measurement began once the chamber pressure reached 2.2 kPa, with a 2 min interval between each measurement.

DSC-TGA Analysis: This analysis was conducted to assess the thermal stability of the synthesized carbons. A SDT Q600 thermogravimetric analyzer (TA Instruments, New Castle, DE, USA) was used. Measurements were carried out under a 100 mL/min flow of Arcal gas (a mixture of 95% Ar and 5% H_2_; Air Liquide, Kraków, Poland) to provide a reducing atmosphere.

MP-AES Analysis: The efficiency of Ni adsorption was evaluated using microwave plasma atomic emission spectroscopy (MP-AES). An Agilent 4210 MP-AES analyzer (Agilent Technologies, Santa Clara, CA, USA) was used. Calibration was performed using the ICP multi-element standard solution IV(Merck KGaA, Darmstadt, Germany) at concentrations of 1, 2.5, 5, 10, 25, and 50 ppm. Calibration solutions were prepared by cascade dilution using 1 M HNO_3_ as the diluent. For analysis, 1 mL of each filtrate was diluted with water to obtain 10-fold and 100-fold diluted samples. In total, six test samples and one reference (designated as “S”) were prepared.

XRD Analysis: X-ray diffraction analysis was conducted to verify the composition of the obtained materials. A Rigaku MiniFlex II Desktop Powder X-ray Diffractometer (Rigaku, Tokyo, Japan) was used for this purpose.

SEM-EDS Study: This study was performed to examine the surface morphology of the carbon materials and to assess the distribution of Ni nanoparticles on the supports. The specimens were observed using a JEOL-6000 Plus Scanning Electron Microscope (JEOL, Tokyo, Japan). Observations were conducted under high vacuum at accelerating voltages ranging from 5 to 15 kV. Magnification varied from 22× to 5000×. The observed Ni-impregnated carbon pellets had a diameter of 10 mm and a height of 7 mm.

Ash Content: Ash content was measured to determine the total mineral content of the obtained carbons. The procedure was the same for all three samples: each carbon sample was placed in a quartz crucible and inserted into a tube furnace (Czylok, Jastrzębie-Zdrój, Poland). The temperature was increased from 19 °C to 800 °C over 12 h in an air atmosphere. The resulting ash was then weighed using an analytical balance.

FT-IR Study: The main functional groups present on the carbon surfaces were identified using a Nicolet 380 FT-IR spectrometer (Thermo Fisher Scientific, Waltham, MA, USA). Spectra were recorded in the range of 4000–500 cm^−1^ with 50 scans at a resolution of 4 cm^−1^ in transmission mode. For sample preparation, 0.2 g of KBr (Merck, Darmstadt, Germany) was mixed with 0.0001 g of activated carbon powder, homogenized in an agate mortar, and pressed into a 10 mm diameter pellet using a hand-operated hydraulic press at a pressure of 10 MPa. A reference sample was prepared in the same manner using pure KBr, and its spectrum was recorded before the analysis of the research samples to provide a background.

## 3. Results

### 3.1. BET Analysis

To determine the values of specific surface area (SSA) of the obtained carbons, BET adsorption–desorption plots were created.

**Figure 1:**
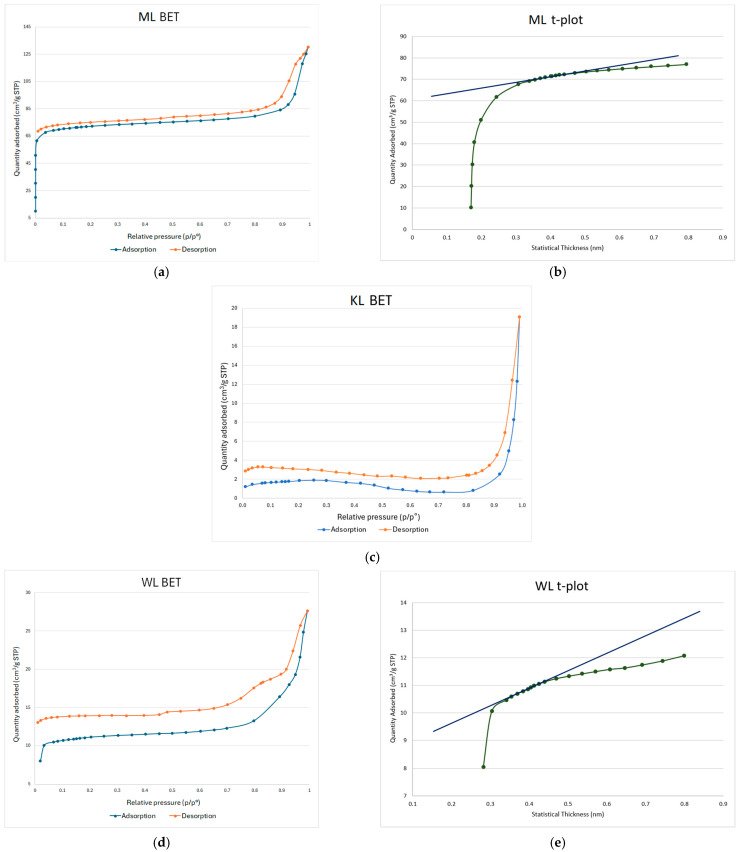
BET isotherms for ML (**a**), KL (**c**), and WL (**d**) carbons, and t-plots for ML (**b**) and WL (**e**) carbons.

In Figure 1, the BET isotherms for carbons obtained from maple (a), knotweed (c), and willow (d) leaves are shown. The shape of the isotherms and the corresponding values align with the specific surface area (SSA) data presented in Table 2. The sample ML exhibits the highest SSA among all the synthesized activated carbons. WL has the second-highest SSA, while KL shows the lowest SSA. From this data, it can be concluded that, among the starting materials used in this study, maple leaves offer the best potential for achieving a high specific surface area. This may be attributed to the degree of fragmentation of the starting materials. Regarding pore distribution, ML carbon exhibits the highest mesopore volume (Table 3), with mesopores being the dominant pore type in this material. A significantly lower mesopore volume is observed in WL carbon (Table 4), while KL shows the lowest porosity, in fact falling below the detection limit of the device used for the BET analysis. A similar trend can be observed in the t-plots. In the case of ML carbon (Figure 1b), the initial upward curvature of the adsorption data (green line) at low thickness indicates micropore filling. The y-intercept of the fitted blue line provides an estimate of micropore volume, which is approximately 0.1 cm^3^/g. The slope of the linear region relates to multilayer adsorption on the external surface, from which the external surface area is calculated to be 40 m^2^/g. For WL carbon (Figure 1e), a slight upward deviation from the linear trend at low statistical thickness (below ~0.35 nm) suggests limited microporosity, likely only trace amounts. The y-intercept of the linear fit is just above zero, indicating a small micropore volume of approximately 0.01 cm^3^/g. The slope of the linear region reflects an external surface area of 10 m^2^/g. Since the t-plot remains mostly linear beyond ~0.35 nm, this suggests that multilayer adsorption dominates, typically for mesoporous or nonporous materials.

### 3.2. DSC-TGA Analysis

DSC-TGA measurements were performed to study the sample masses’ reduction and energetic effects during heating. This allowed the authors to check the effectiveness of pyrolysis and the samples’ thermal stability.

**Figure 2:**
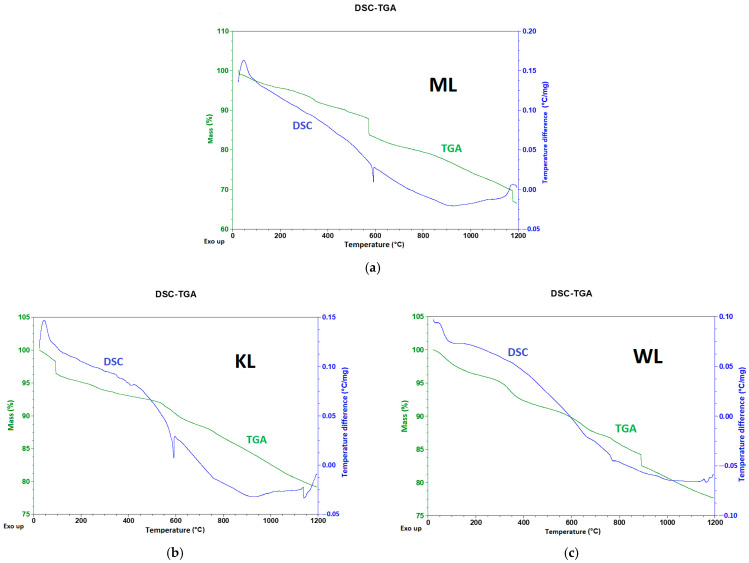
DSC-TGA plots for ML (**a**), KL (**b**), and WL (**c**) Ni-impregnated carbons.

The most significant mass reduction is observed for the ML sample (Figure 2a, green line), reaching 34%. This result likely stems from the fine-grain size of this carbon, which increases the surface area available for heat transfer. As a result, the decomposition of the material during heat treatment is enhanced. The sharp mass loss observed near 600 °C is likely due to the reduction of oxygen-containing surface groups. This is supported by the corresponding signal in the temperature differential plot (blue line), which shows a rapid transition from endothermic to exothermic behavior. In the case of the KL sample (Figure 2b), the total mass reduction (green line) is approximately 21%, significantly lower than that of ML. A sharp mass drop around 600 °C is not present in this sample; however, the endothermic-to-exothermic transition (blue line) still occurs at this temperature. Additionally, a second energetic transition is observed near 1150 °C, suggesting that, at higher temperatures, oxygen groups located in deeper layers of the carbon structure may also undergo reduction. For the WL carbon (Figure 2c), the behavior differs notably. A sharp mass drop similar to that seen in ML occurs but at a much higher temperature—around 900 °C (green line). The total mass loss for this sample is 23%, only slightly higher than that of KL. An energetic shift (blue line) is also observed, but it takes place near 800 °C and is less pronounced compared to the transitions in the other two samples. This indicates that WL carbon exhibits the highest thermal stability among the three materials tested.

### 3.3. MP-AES Analysis

MP-AES analysis was performed to check Ni concentration in the post-impregnation solutions. Then, the authors compared the results with a starting Ni solution, which made it possible to calculate the amount of Ni adsorbed on each carbon sample.

The MP-AES analysis revealed that ML exhibited the highest nickel adsorption capacity: 1 g of this material adsorbed 165 mg of Ni (Table 5). The lowest adsorption was observed for KL. These results are consistent with the findings of the BET study: the carbon with the largest specific surface area (SSA) adsorbed the most nickel, while the carbon with the smallest SSA adsorbed the least. However, when comparing the areal concentration of nickel (i.e., the amount of nickel per unit surface area), the trend is reversed. The KL carbon exhibits the highest areal Ni concentration—nearly 16 mg/m^2^—which is over five times greater than that of WL. In contrast, ML reaches only 1 mg of Ni per 1 m^2^ of surface area. This may suggest that KL carbon possesses the highest density of functional groups capable of binding nickel ions. These organic surface groups likely enhance the material’s affinity for Ni, enabling effective adsorption despite a relatively low overall surface area.

### 3.4. XRD Analysis

To determine the crystallographic phases and confirm the results from the MP-AES study, the authors conducted XRD measurements of the Ni-impregnated carbons. 

**Figure 3:**
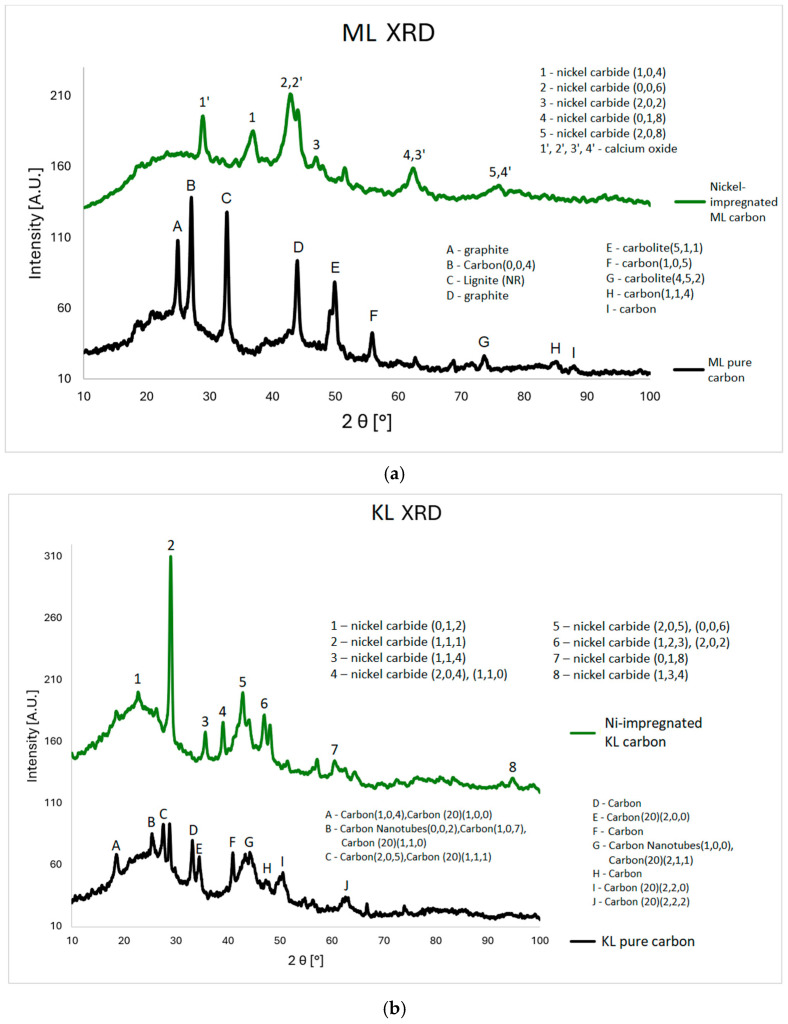

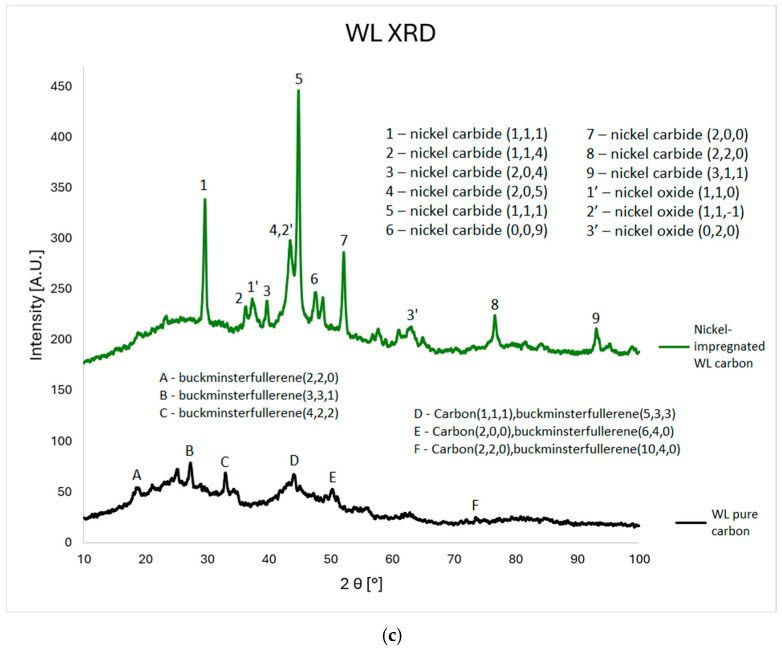
XRD plots for ML (**a**), KL (**b**), and WL (**c**) Ni-impregnated activated carbon samples and corresponding pure carbons for comparison.

In this study, a series of material data sheets were used; their reference numbers can be found in the Supplementary Materials. For the Ni-impregnated ML carbon (Figure 3a), five peaks corresponding to nickel carbide and four peaks related to calcium oxide are observed. In contrast, the non-impregnated ML sample exhibits two peaks associated with graphite, four with carbon, and two with carbolite. In the case of the Ni-impregnated KL carbon (Figure 3b), eight peaks corresponding to nickel carbide are identified. The non-impregnated KL sample shows ten carbon-related peaks (including three from amorphous carbon) and two peaks from carbon nanotubes. For the Ni-impregnated WL carbon (Figure 3c), nine peaks from nickel carbide and three from nickel oxide are present. The non-impregnated WL carbon shows six signals from buckminsterfullerene and three from carbon. These findings align with the results obtained from the MP-AES and SEM-EDS analyses, particularly for the ML and WL samples. In the EDS elemental maps, distinct deposits of Ni, Ca, and O are visible. Moreover, the spatial overlap of Ca and O, as well as Ni and O, supports the presence of calcium oxide and nickel oxide, respectively.

### 3.5. SEM-EDS Study

To check the samples’ topography and distribution of Ni and other elements across the samples’ surfaces, SEM observations with EDS analysis were conducted.

**Figure 4:**
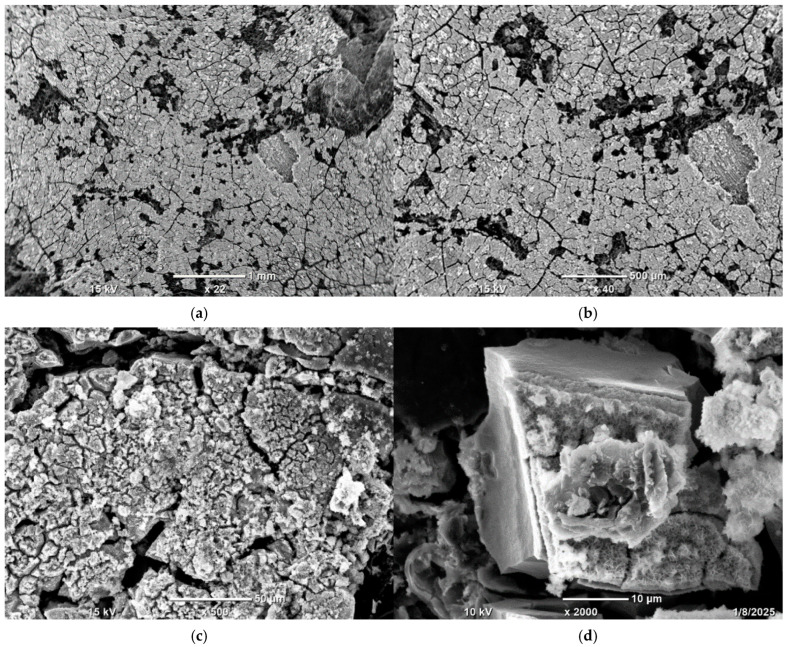
SEM photographs of ML Ni-impregnated activated carbon at 22 times (**a**), 40 times (**b**), 500 times (**c**) and 2000 times (**d**) magnification.

**Figure 5:**
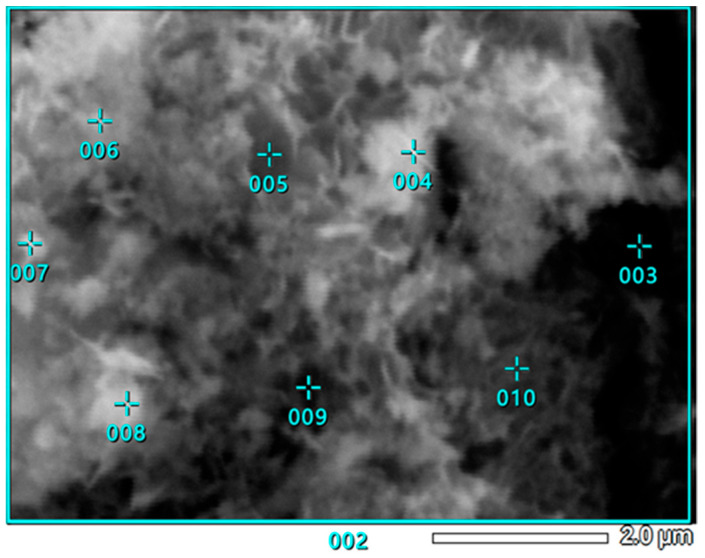
SEM photograph of Ni-impregnated ML activated carbon’s surface covered with measurement points for EDS analysis.

In Figure 4, a well-developed surface of Ni-impregnated ML activated carbon can be observed. By examining Figure 5 and Table 6, it is evident that the highest percentage of Ni is found at point 003, while the lowest concentration is observed at point 009. Aside from these two extremes, a gradient in Ni concentration is visible across the remaining points, decreasing from the upper left corner (higher values) to the lower right corner (lower values). This distribution suggests that areas with a more complex surface structure are more likely to adsorb larger amounts of nickel, likely due to an increased number of active sites or favorable surface chemistry.

**Figure 6:**
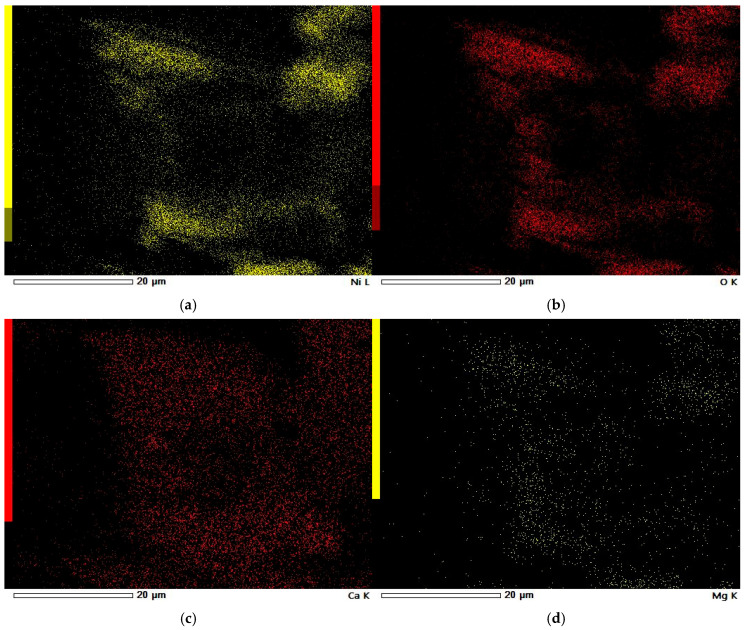
EDS elemental maps of Ni-impregnated ML activated carbon: Ni (**a**), O (**b**), Ca (**c**), and Mg (**d**), corresponding to SEM photograph from Figure 4d.

In Figure 6a–d, a clear pattern can be observed. Firstly, the highest concentration of metal deposits appears at the edges of the carbon grains, indicating that these regions exhibit the strongest adsorption capability. Secondly, there is a notable overlap in the distribution of oxygen, nickel, calcium, and magnesium, suggesting that these metals are present in the sample both as oxides and as elemental (metallic) forms.

**Figure 7:**
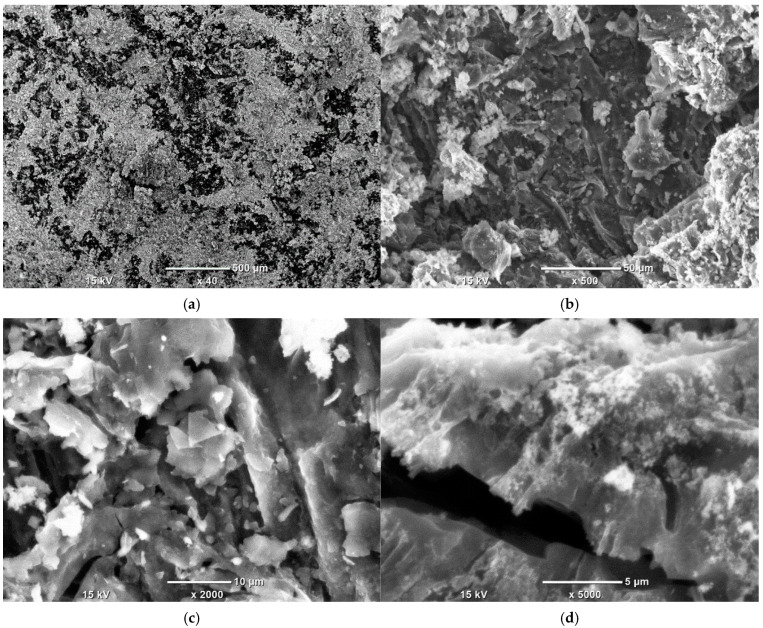
SEM photographs of Ni-impregnated KL activated carbon’s surface at 40 times (**a**), 500 times (**b**), 2000 times (**c**) and 5000 times (**d**) magnification.

**Figure 8:**
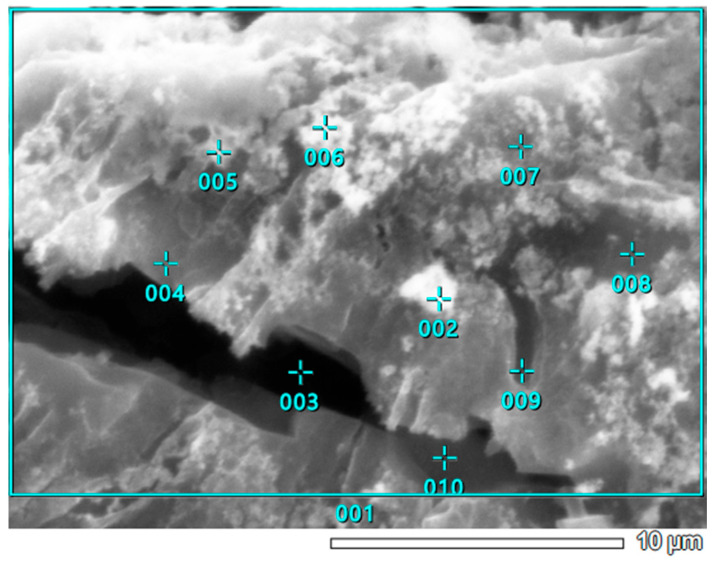
Point EDS analysis of Ni-impregnated KL activated carbon.

In Figure 7, the well-developed surface of the KL sample can be observed. According to Table 7 and Figure 8, the highest nickel concentration is located at the large white spot where point 002 is placed. The lowest concentration of Ni is found at point 007, which is situated farthest from the edge of the activated carbon grain. Interestingly, point 004, despite being closest to the grain’s edge, also shows a low Ni content, only slightly higher than at point 007. Apart from point 002, elevated Ni concentrations are also observed at points 005 and 006, suggesting that nickel tends to accumulate in intermediate surface regions rather than exclusively at the edges or centers. Regarding alkali metals, the highest magnesium concentration is found at points 008 and 009, whereas the highest calcium and potassium levels are observed at point 003, located at the grain’s edge. This distribution indicates that alkali metals do not co-localize; the presence of one generally excludes the presence of the others in the same region.

**Figure 9:**
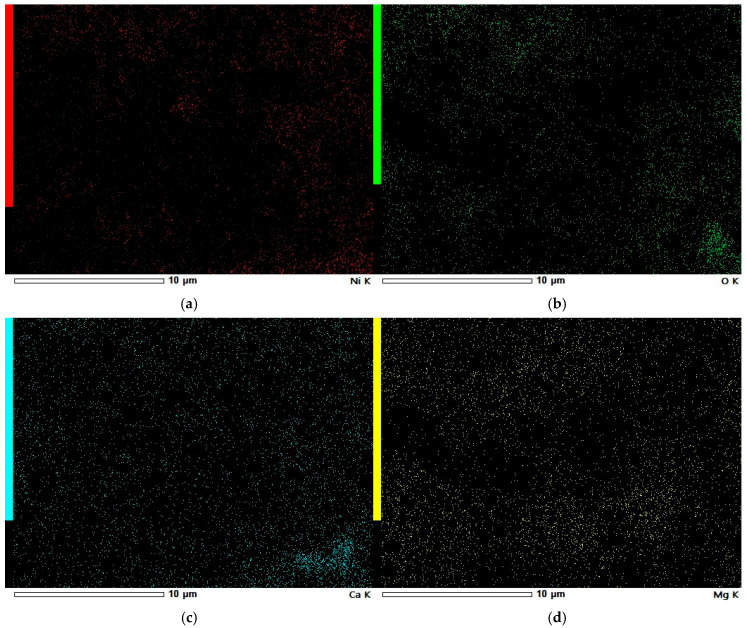
EDS elemental maps of Ni-impregnated KL activated carbon: Ni (**a**), O (**b**), Ca (**c**), and Mg (**d**).

In Figure 9a, it can be seen that the KL carbon adsorbs significantly less nickel than the ML carbon, although the distribution of nickel across the KL surface remains relatively uniform. On the other hand, KL shows a higher content of alkali metals. In Figure 9c, a region of calcium agglomeration is visible, indicating that calcium tends to form larger aggregates on the surface of KL compared to other metals. Notably, the location of this calcium-rich area corresponds to a region of oxygen deposition observed in Figure 9b. This supports the conclusion that calcium is present in the form of an oxide on the surface of the carbon.

**Figure 10:**
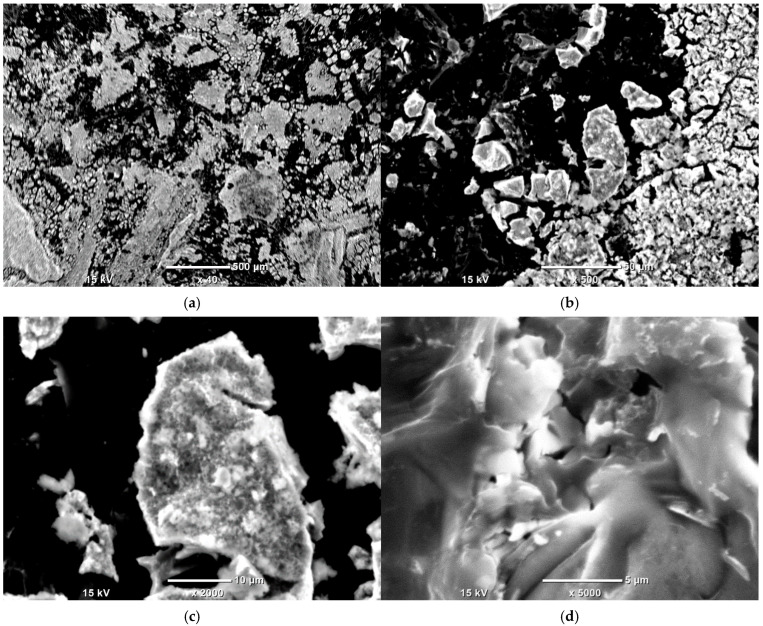
SEM photographs of the Ni-impregnated WL activated carbon sample at 40 times (**a**), 500 times (**b**), 2000 times (**c**) and 5000 times (**d**) magnification.

**Figure 11:**
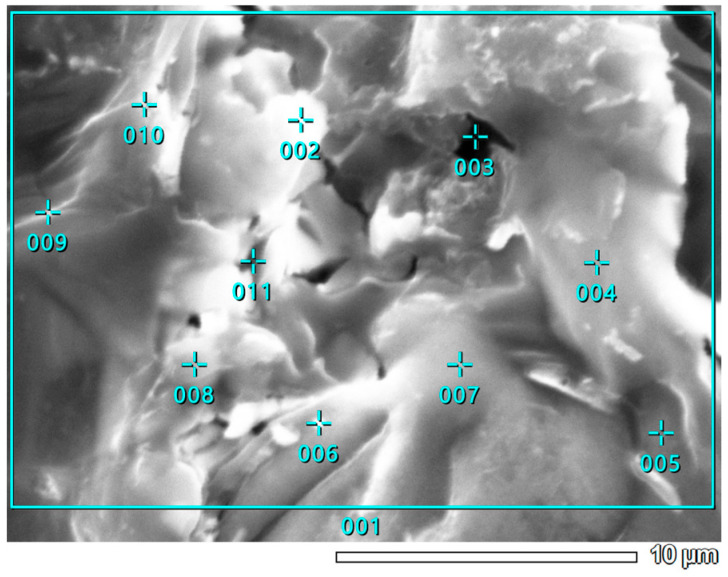
Point EDS analysis of Ni-impregnated WL activated carbon from Figure 10d.

**Figure 12:**
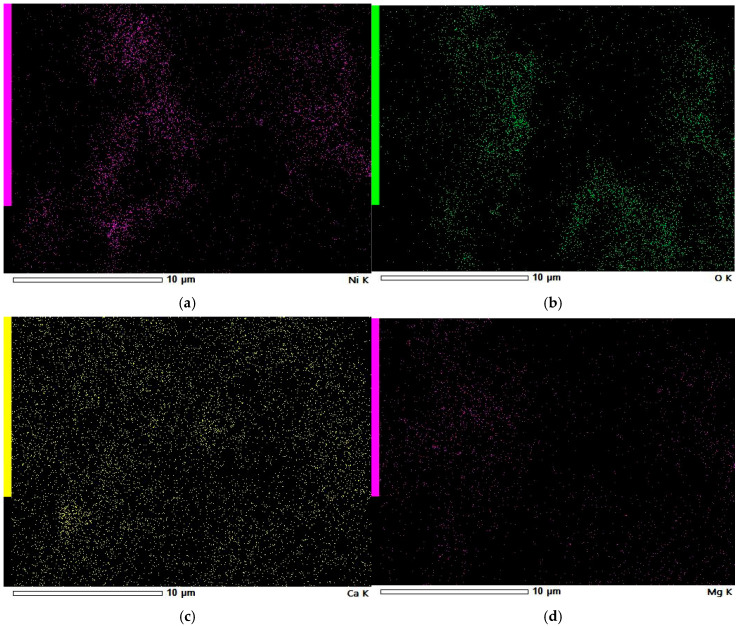
EDS elemental maps of Ni-impregnated WL activated carbon: Ni (**a**), O (**b**), Ca (**c**), and Mg (**d**).

Figure 10 illustrates the surface morphology of the WL carbon. According to Table 8, the highest nickel concentration is found at point 003, where the Ni content exceeds 55%. This point also shows the highest concentrations of potassium and calcium. In contrast, at point 009, the nickel content is below the detection limit, although notable amounts of alkali metals, including magnesium, are still present. Furthermore, the areal quantitative EDS results, represented by area 001 for samples KL (Figure 8; Table 7), WL (Figure 11; Table 8), and area 002 for ML (Figure 5; Table 6), align well with the findings from the MP-AES study. Specifically, the ML sample shows a total Ni concentration of over 26%, WL has just over 7%, and KL remains below 5%. These values accurately reflect the nickel adsorption capacity of each carbon material. Therefore, it can be concluded that the results of the qualitative EDS analysis (elemental maps from Figure 12) are consistent with the quantitative point analysis.

Based on the EDS elemental maps of WL shown in Figure 12, it can be observed that the surface is covered with nickel particles, although their distribution is irregular. These nickel deposits are concentrated in specific areas, many of which overlap with oxygen-rich regions. This indicates that nickel primarily exists as nickel oxide on the WL carbon surface. A similar pattern is observed for magnesium, which also appears to be present mainly in oxide form. In contrast, calcium is more uniformly distributed across the surface, suggesting that it exists in both crystalline and oxide forms on the WL carbon.

### 3.6. Ash Content

To check the total amount of mineral ingredients in the carbons, their ash content was examined.

The highest ash content (Table 9) was found in the ML carbon, reaching close to 28%, which is a value quite similar to that of the 2p carbon series studied in the previous work [35]. The WL carbon contained a significantly lower amount of mineral components at 20%, while the KL carbon showed the lowest level of these substances, at only 19%.

### 3.7. FT-IR Study

To check the types of functional groups occurring on the carbons’ surfaces, an FT-IR analysis for each sample was conducted.

**Figure 13:**
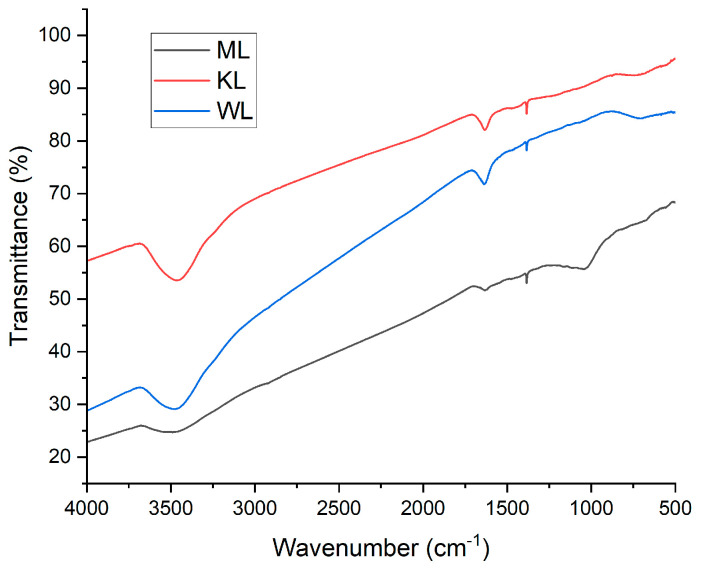
FT-IR spectra of Ni-impregnated ML, KL, and WL activated carbons.

As shown in Figure 13, all the studied carbons exhibit a set of distinctive spectral features. The broad band between 3250 and 3750 cm^−1^ corresponds to physically adsorbed water and arises from the stretching of the O–H bond. The broad peak at 1650 cm^−1^ is attributed to ketone surface groups, resulting from the stretching of the C=O bond. This functional group is also present in lactones, which produce a relatively small but sharp peak at 1350 cm^−1^. Around 700 cm^−1^, there is another broad band caused by the asymmetric bending deformation of the C–H_2_ group. Only in the case of ML is an additional band observed in the range of 750–1250 cm^−1^. This band is due to the stretching of the C–O bond in ethers, lactones, and phenols [36,37,38,39].

## 4. Discussion

Looking at the BET figures (Figure 1), it can be seen that the adsorption and desorption plot lines are not superimposed. In all three cases, the desorption line lies below the adsorption line. This displacement is called a hysteresis loop, and its occurrence indicates the presence of mesopores in the synthesized carbons. Mesopores are desirable because they increase the material’s specific surface area (SSA), providing a high number of adsorption sites for Ni. Mesopores are preferred over micropores because, although micropores improve the effectiveness of metal ion adsorption, they can trap gas particles.

In all three DSC-TGA graphs (Figure 2), an initial mass drop can be observed at the beginning of each plot, caused by the evaporation of residual water. A particularly drastic mass reduction is visible in the KL plot (Figure 2b) near 100 °C. Between 200 °C and 600 °C, the mass loss slows down, likely representing the decomposition of cellulose and lignin. The WL sample (Figure 2c) is an exception; here, the mass reduction curve is more irregular compared to the other two carbons, forming characteristic “waves” up to 900 °C, where another steep drop occurs. This pattern may arise from the material’s more complex chemical composition, causing the thermal decomposition process to occur in multiple stages. Each change in the curve’s slope corresponds to the evaporation of a different fraction of organic compounds. Considering the temperature change plots (blue lines), WL shows the most regular shape (Figure 2c). ML (Figure 2a) and KL (Figure 2b) carbons exhibit distinct “cracks” near 600 °C, which represent rapid transitions from endothermic to exothermic processes and back again. This temperature likely corresponds to significant structural changes and the reduction of oxygen deposits, indicating limited thermal stability under harsh heat conditions.

The values obtained from the MP-AES study (Table 5) confirm the successful impregnation process and clearly illustrate the Ni adsorption capacity of each material. As expected, the highest amount of Ni was adsorbed on ML carbon, which has the largest SSA. The WL sample showed a much lower Ni adsorption, while the KL carbon adsorbed the least. The gap between ML and WL is approximately six times larger than that between WL and KL. This difference likely originates from the properties of the starting materials. The dried ground maple leaves have a very fine dust-like consistency, dried and ground willow leaves resemble ground pepper, and ground knotweed leaves have a texture similar to bagged tea. Therefore, even before pyrolysis, ML has a more developed surface than the other two materials, and carbonization further enhances this feature. An interesting observation from the last column of Table 5 is that the KL carbon, despite having the lowest Ni amount per gram, exhibits the highest areal Ni density; that is, the amount of Ni distributed per 1 m^2^ of surface area is more than five times greater than WL and about sixteen times higher than ML. This suggests that KL has the highest density of accessible functional groups that attract and bind nickel ions. Numerous studies describe the Ni–C bonding mechanisms [40,41,42,43,44], showing that bonding depends on factors such as the chemical nature and structure of the carbon support, the pH of the impregnation solution, and, in the case of biomass-derived carbons, the types of functional groups present on the surface. According to the literature, the most effective functional groups contain oxygen and nitrogen, such as –CO, –C–O, –COO–, or –NH–.

The XRD results (Figure 3) confirm the presence of nickel in the impregnated samples. Nickel appears mainly in two forms: metallic crystalline Ni and nickel carbide. Both forms exhibit multiple crystalline phases, indicated by peaks at specific angles corresponding to distinct hkl indices. The spectra also show calcium’s presence, likely due to the precursor material originating from a post-industrial area where calcium in the soil may be a remnant of building materials. This calcium was subsequently accumulated in the plant leaves. Unlike Ni, calcium appears only as calcium oxide.

The SEM observations enabled a detailed examination of the samples’ surface topography (Figure 4, Figure 7 and Figure 10) and elemental distribution mapping (Figure 6, Figure 9 and Figure 12). The results show good Ni distribution across all three materials. Alkaline earth metals (Mg and Ca) are also present and well dispersed. The oxygen presence originates from organic groups in the pyrolyzed material. Additionally, the working atmosphere may not have been completely inert: trace oxygen in the nitrogen gas could have led to partial oxidation of the carbon surfaces.

The ash content study revealed that the carbons synthesized in this work differ from typical industrial activated carbons (ACs). These materials contain a very high mineral content, ranging from 19% to 28% (Table 9), compared to approximately 7% in commercial ACs. Although higher ash content reduces the carbon fraction, it indicates a successful pyrolysis process where most organic compounds were removed, leaving predominantly free carbon in the AC.

The FT-IR measurements (Figure 13) helped to identify the functional groups present on the material surfaces. Most of the detected groups are residues of lignocellulosic biomass and its decomposition products. The data also suggest the types of bonds that may form between the carbon surfaces and Ni ions. The abundant oxygen-containing groups on the ACs imply that Ni is bonded via coordinate bonds, with oxygen atoms acting as electron donors and Ni cations as acceptors.

For a better assessment of the obtained materials’ properties, comparable systems from other studies were included for reference (Table 10).

The leaves of goldenrod have the highest mineral content among all the compared materials, with maple leaves showing only a slightly lower value. Willow and knotweed leaves exhibit much lower ash content, with reed having the lowest, roughly five times less mineral content than maple leaves. Regarding fixed carbon content, the materials obtained in this study outperform carbons derived from goldenrod and reed. In terms of Ni loading, ML carbon has the highest value among all the specimens. Willow- and knotweed-based activated carbons (ACs) adsorbed about 1.5 times less Ni, while the goldenrod-based carbon showed the poorest performance, with over twice less Ni per gram of support compared to ML. However, the trend reverses when looking at the Ni surface distribution: goldenrod-derived carbon has the highest Ni areal density, KL shows a significantly lower Ni-to-surface ratio, and all the other carbons have less than 1 mg of Ni per square meter of surface area. These differences are related to plant morphology, which depends on their growth habitat. For example, reed grows in water or wet soil, resulting in a higher moisture content than plants typically found in dry environments. This leads to lower organic compound content in reed, creating a looser structure with a larger SSA but fewer functional groups per square meter. The low ash and fixed carbon contents in reed also stem from its higher water content, which is lost during drying and pyrolysis. The variations in surface development among ML, KL, and WL primarily reflect physical differences in the starting materials. Dried maple leaves can be ground into the finest particles among these three, while dried willow leaves have the lowest fragmentation level. This difference affects the heat distribution during pyrolysis, influencing the degree of material decomposition, resulting particle size, and surface topology. The SEM images clearly show that ML carbon has the most cracks and pores on its surface, while the textures of the other carbons are less complex.

## 5. Conclusions

In this study, a series of nickel–carbon composites were synthesized using leaves from different plant species. The materials were characterized by various analytical techniques. The ML carbon exhibited the highest specific surface area (SSA) of 266 m^2^/g and the greatest nickel adsorption capacity (165 mg Ni per 1 g of carbon). The WL carbon demonstrated the best thermal stability, a uniform nickel distribution across its surface, and the highest content of alkali metals. The KL carbon showed the highest nickel areal density at 16 mg/m^2^. Among the samples, ML contained the highest amount of mineral content, while KL had the lowest. ML also displayed the highest porosity and the greatest number of mesopores. In contrast, KL’s porosity was below the detection limit, yet it still exhibited a respectable nickel adsorption capacity. According to the literature, these materials show potential for successful implementation as catalysts in dye oxidation [46], hydrogen evolution [47], hydrogenation [48], or methane decomposition [49]. However, the results suggest that their most suitable application is a catalytic CO_2_ conversion to methane [45,50,51].

## Figures and Tables

**Table 1:** Masses of starting plant materials and obtained carbons with their yield.

Material/Parameter	ML	KL	WL
Carbon yield [%]	31	31	30
Mass before pyrolysis [g]	14.9825	13.7561	16.0704
Mass after pyrolysis [g]	4.6474	4.2604	4.8317

**Table 2:** SSA values for ML, KL, and WL activated carbons.

Sample Name	ML	KL	WL
Specific surface area [m^2^/g]	266	7	40

**Table 3:** BET pore distribution in ML activated carbon.

dV/dlog (w) Pore Volume (cm^3^/g)	Average Width (nm)	Incremental Pore Volume (cm^3^/g)	Cumulative Pore Volume (cm^3^/g)	Incremental Pore Area (m^2^/g)	Cumulative Pore Area (m^2^/g)
2.2796 × 10^−2^	181.2	0.0075	0.0075	0.2	0.2
4.0255 × 10^−2^	88.8	0.0124	0.0199	0.6	0.7
1.2942 × 10^−1^	43.7	0.0397	0.0595	3.6	4.4
9.3578 × 10^−2^	29.5	0.0139	0.0734	1.9	6.2
5.3035 × 10^−2^	21.7	0.0068	0.0802	1.3	7.5
2.9315 × 10^−2^	12.4	0.0077	0.0879	2.5	10.0
1.3922 × 10^−2^	8.1	0.0024	0.0903	1.2	11.1
1.0244 × 10^−2^	6.5	0.0008	0.0911	0.5	11.6
9.4051 × 10^−3^	5.6	0.0006	0.0916	0.4	12.0
9.7029 × 10^−3^	4.9	0.0006	0.0922	0.5	12.5
8.8048 × 10^−3^	4.3	0.0005	0.0926	0.4	12.9
9.3416 × 10^−3^	3.8	0.0005	0.0931	0.5	13.4
1.3367 × 10^−2^	3.4	0.0007	0.0938	0.8	14.2
1.6423 × 10^−2^	3.0	0.0008	0.0946	1.0	15.2
1.6695 × 10^−2^	2.7	0.0008	0.0953	1.1	16.3
2.2995 × 10^−2^	2.5	0.0011	0.0965	1.8	18.2
2.8047 × 10^−2^	2.2	0.0013	0.0978	2.4	20.6

**Table 4:** BET pore distribution in WL activated carbon.

dV/dlog (w) Pore Volume (cm^3^/g)	Average Width (nm)	Incremental Pore Volume (cm^3^/g)	Cumulative Pore Volume (cm^3^/g)	Incremental Pore Area (m^2^/g)	Cumulative Pore Area (m^2^/g)
9.8839 × 10^−3^	109.2	0.0046	0.0046	0.2	0.2
2.8576 × 10^−2^	67.8	0.0056	0.0102	0.3	0.5
2.2894 × 10^−2^	45.0	0.0040	0.0141	0.4	0.9
1.5178 × 10^−2^	31.3	0.0023	0.0165	0.3	1.1
1.8405 × 10^−2^	21.8	0.0029	0.0193	0.5	1.7
2.3890 × 10^−2^	12.2	0.0064	0.0257	2.1	3.8
8.1924 × 10^−3^	8.0	0.0014	0.0272	0.7	4.5
2.4600 × 10^−3^	6.4	0.0002	0.0273	0.1	4.6
1.8533 × 10^−3^	5.5	0.0001	0.0274	0.1	4.7
1.3253 × 10^−4^	4.8	0.0000	0.0275	0.0	4.7
4.0215 × 10^−4^	2.2	0.0001	0.0276	0.2	4.9

**Table 5:** Ni concentrations in filtrates after impregnation of carbons with a 63 mM (3035 ppm) solution of Ni(NO_3_)_2_·6H_2_O. The “S” sample was used as a reference; it was a part of the starting Ni solution, not used for carbon impregnation.

Filtrate Name/Parameter	ML	KL	WL	S
Amount of Ni per 1 g of carbon [mg/g]	165	15	117	-
Amount of Ni per 1 m^2^ of carbon’s specific surface [mg/m^2^]	0.6	15.9	0.9	-
Ni concentration in the filtrate after impregnation [mmol/L]	21	37	33	63

**Table 6:** Ni and other elements’ atomic concentration [%] at measurement points shown in Figure 5.

Point Number	Chemical Element Concentration [Atomic %]								
	C	O	Si	S	K	Ca	Ni	Te	Total
001		64.42		1.74		3.15	30.69		100.00
002	10.04	58.26	0.84	0.96	0.52	2.90	26.47		100.00
003	4.98	35.67		1.11	0.66	3.44	54.14		100.00
004	8.58	61.47	0.91	0.98	0.41	2.84	24.81		100.00
005		59.93	0.93	1.21	0.48	3.70	33.23	0.52	100.00
006		42.57	0.69	1.26	0.53	3.85	51.09		100.00
007		43.80	0.80	1.01	0.84	3.84	49.70		100.00
008	9.59	57.77	0.68	0.82	0.60	3.14	27.39		100.00
009	10.69	63.20	0.83	0.90	0.37	2.90	21.11		100.00
010									0.00

**Table 7:** Ni and other elements’ atomic concentration [%] at measurement points shown in Figure 8.

Point Number	Chemical Element Concentration [Atomic %]											
	C	O	Mg	Al	Si	S	Cl	K	Ca	Ni	Rb	Total
001	70.25	19.66	2.19			0.16	0.31	1.18	1.61	4.63		100.00
002	57.67	16.55	1.58	0.31	1.07	0.57	0.25	0.84	3.29	17.87		100.00
003	80.55		1.73				1.00	5.68	7.32	3.72		100.00
004	73.29	18.86	2.61				0.26	1.77	1.52	1.62	0.06	100.00
005	59.20	26.30	1.64	0.15	0.29	0.30	0.17	0.75	1.24	9.96		100.00
006	57.48	29.14	1.25	0.31	0.49	0.27	0.09	0.66	1.63	8.69		100.00
007	74.72	19.29	2.36				0.27	1.08	1.07	1.23		100.00
008	63.84	21.12	4.77					3.45	3.51	3.31		100.00
009	62.34	21.30	4.47					3.43	4.45	4.01		100.00
010	74.13	17.35	2.15		0.25	0.16		1.12	1.97	2.38		100.00

**Table 8:** Ni and other elements’ atomic concentration [%] at measurement points shown in Figure 11.

Point Number	Chemical Element Concentration [Atomic %]												
	C	O	Mg	Si	P	S	Cl	K	Ca	Ni	Zn	Mo	Total
001	59.84	22.88	0.85	3.79	0.28	0.87		1.24	3.13	7.14			100.00
002	41.27	39.49		1.72		2.31		0.63	2.99	11.59			100.00
003	21.95			4.28		3.11		3.24	11.72	55.71			100.00
004	63.38	20.69		3.06		0.91		1.06	1.74	9.16			100.00
005	51.24	24.20		17.29		0.22		3.03	1.25	0.51	2.27		100.00
006	47.16	15.89		13.41	0.42	1.02		3.14	2.88	13.07	2.55	0.46	100.00
007	39.95	43.49		11.23	0.16	0.12		2.11	0.98	0.43	1.53		100.00
008	63.03	11.94	1.21	1.35		1.89		0.96	3.88	15.73			100.00
009	70.98	19.65	2.27		0.40		0.47	1.37	4.87				100.00
010	63.42	24.69	1.66		0.16	0.16	0.18	0.55	2.91	5.63			100.00

**Table 9:** Ash content values for the obtained activated carbons.

Sample Name/Parameter	ML	KL	WL
Carbon mass [g]	0.656	0.626	0.599
Ash mass [g]	0.186	0.118	0.120
Ash content [%]	28.4	18.9	20.0

**Table 10:** Comparison of five Ni-impregnated activated carbons derived from different plants: ML, KL, and WL from this study, the 2p from the previous work [35], and the reed-based FWB700 catalyst [45].

Carbon Name/ Parameter	ML (Maple)	KL (Knotweed)	WL (Willow)	2p (Goldenrod)	FWB700 (Reed)
Specific surface area [m^2^/g]	266	7	40	3	355
Ni mass per 1 g of carbon [mg]	165	105	117	76	155
Ni mass per surface unit [mg/m^2^]	0.6	15.9	0.9	22.4	0.4
Fixed carbon [%]	29.8	30.1	29.3	19.6	14.4
Ash [%]	28.4	18.9	20.0	29.7	6.2
Volatile compounds [%]	69	69	69	72	79

## Data Availability

The original contributions presented in this study are included in the article and Supplementary Materials. Further inquiries can be directed to the corresponding authors.

## Supplementary Materials

The following supporting information can be downloaded at https://www.mdpi.com/article/10.3390/ma18163784/s1.
