# Supplementary material for: The Influence of Starting Plant Material on Ni@C-Type Composites’ Characteristics
Source: Materials (Basel). 2025 Aug 12;18(16):3784. doi: 10.3390/ma18163784 (PMC12387519; doi:10.3390/ma18163784)
Supplement: Supplementary file 1 [file materials-18-03784-s001.zip › materials-3763052-supplementary.pdf]

Maple leaves

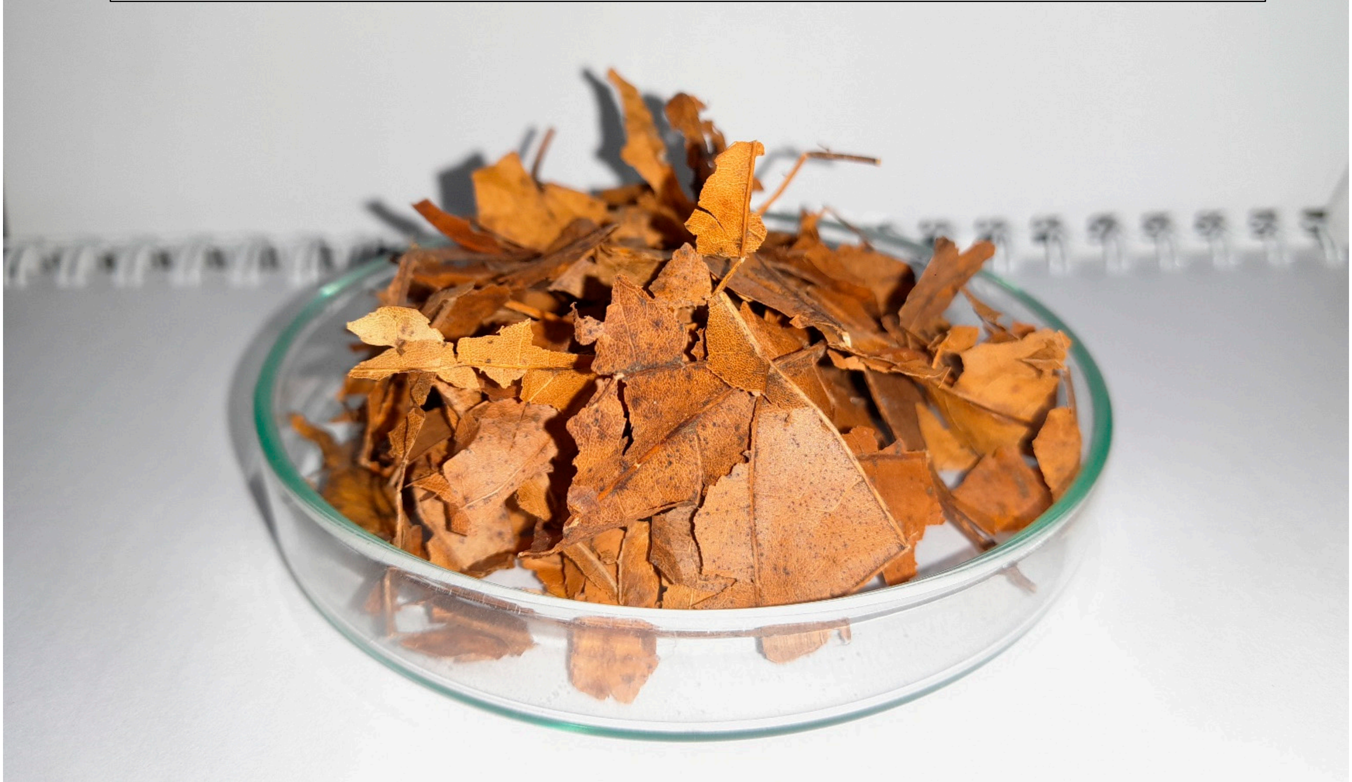

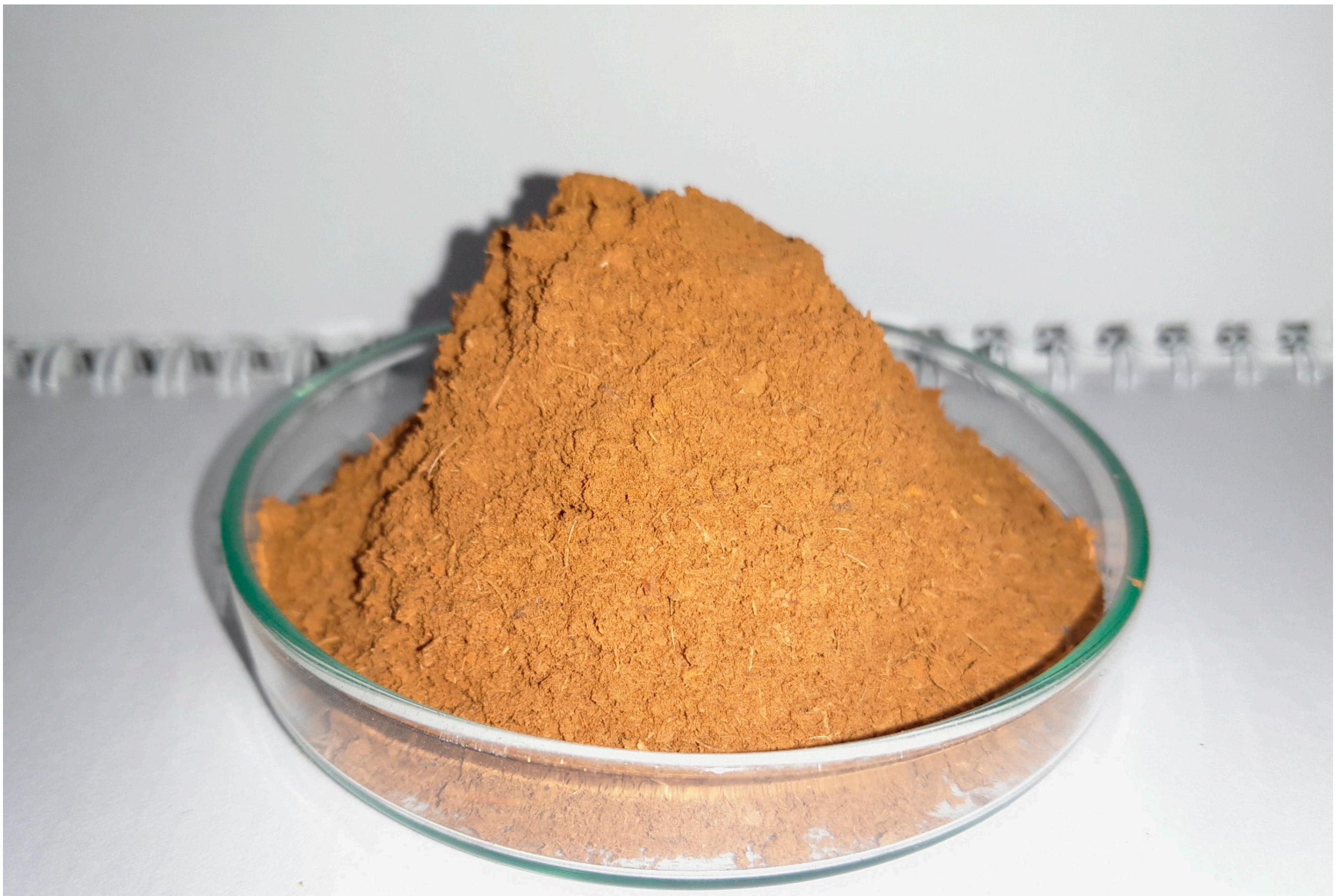

Ground maple leaves

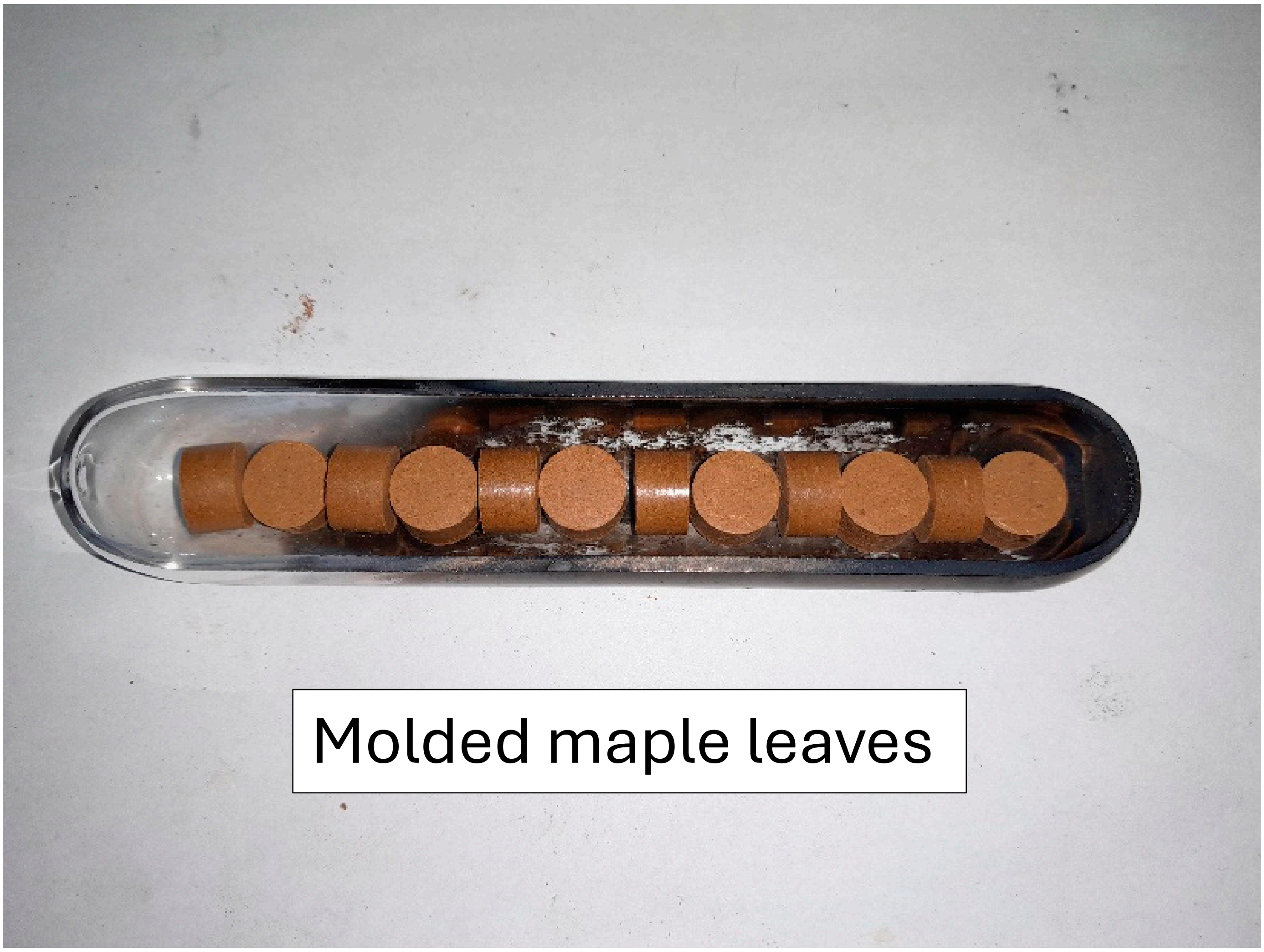
Molded maple leaves

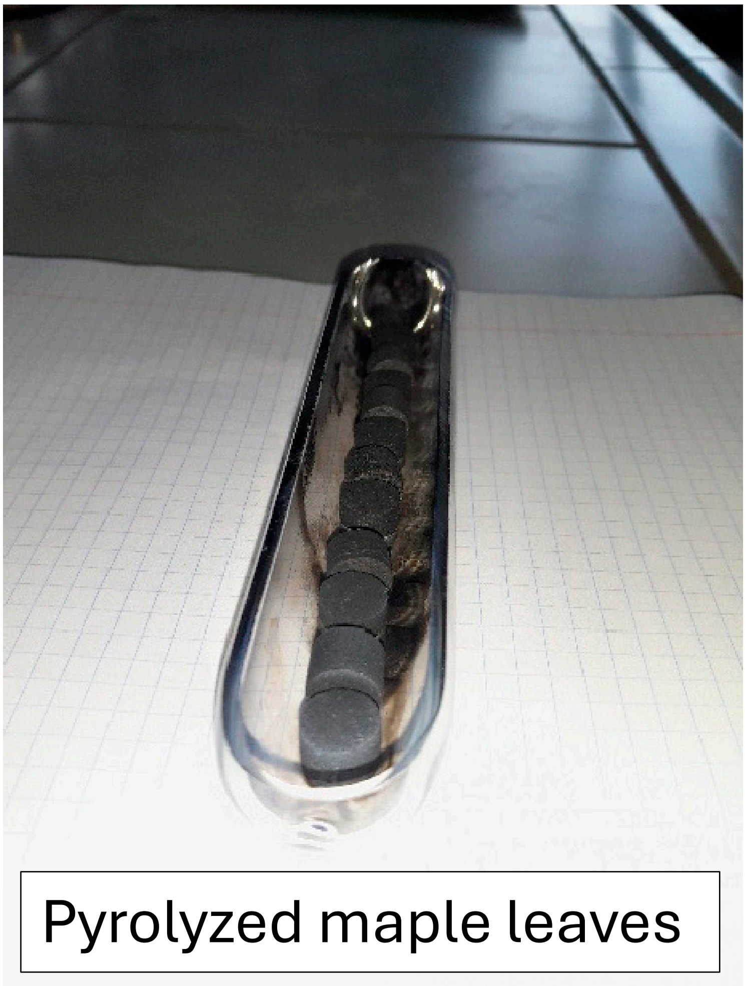
Pyrolyzed maple leaves

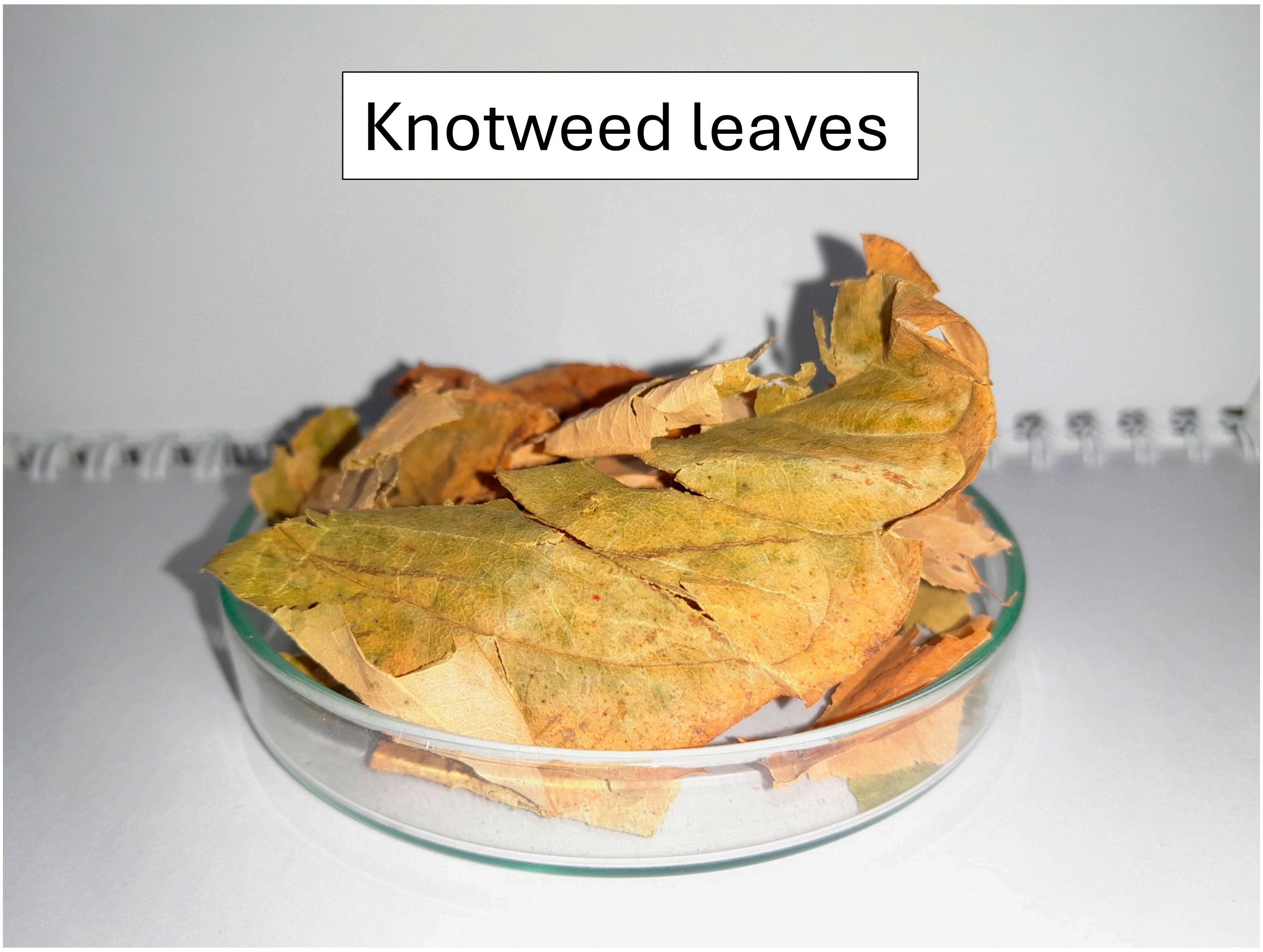
Knotweed leaves

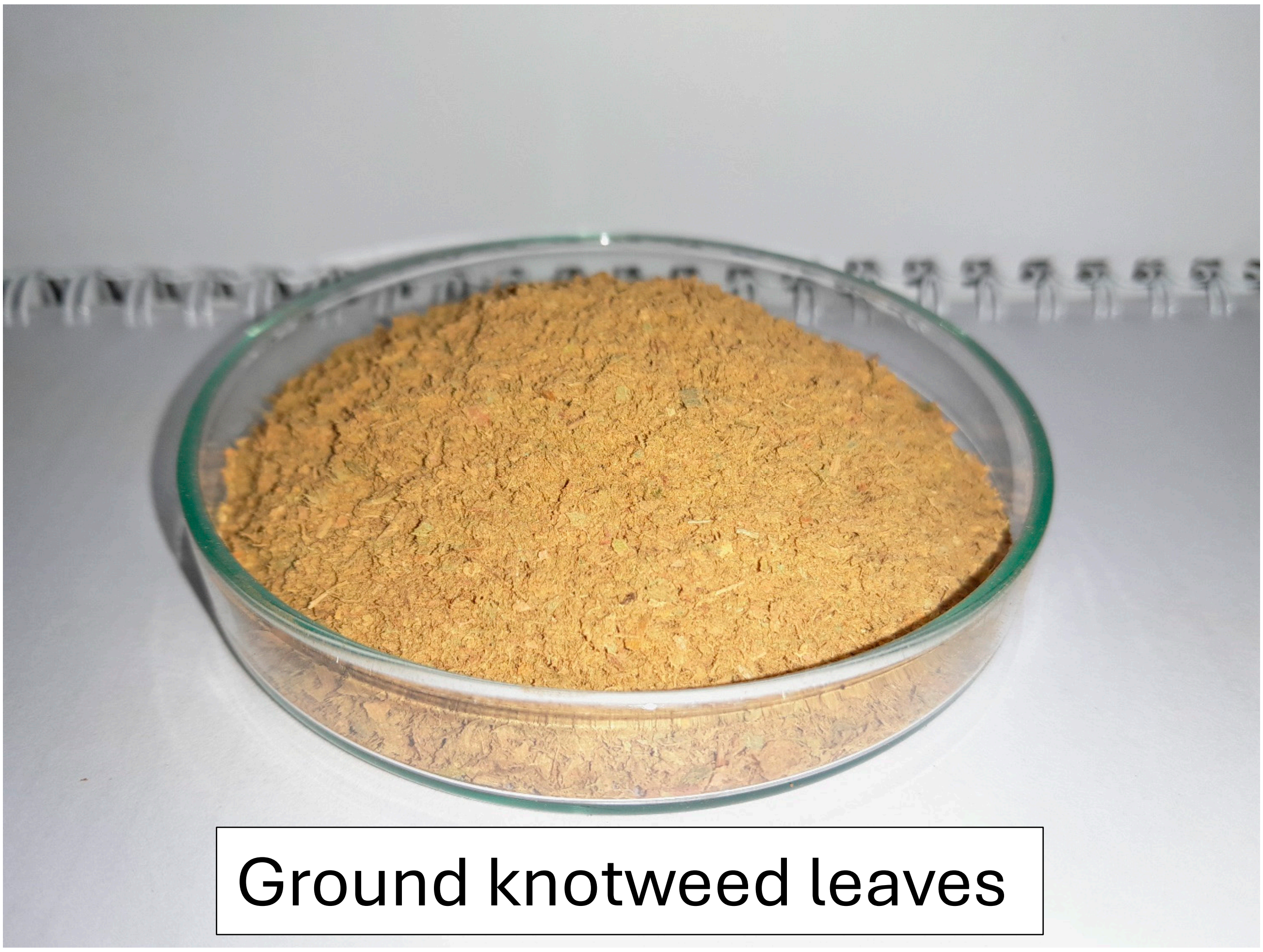
Ground knotweed leaves

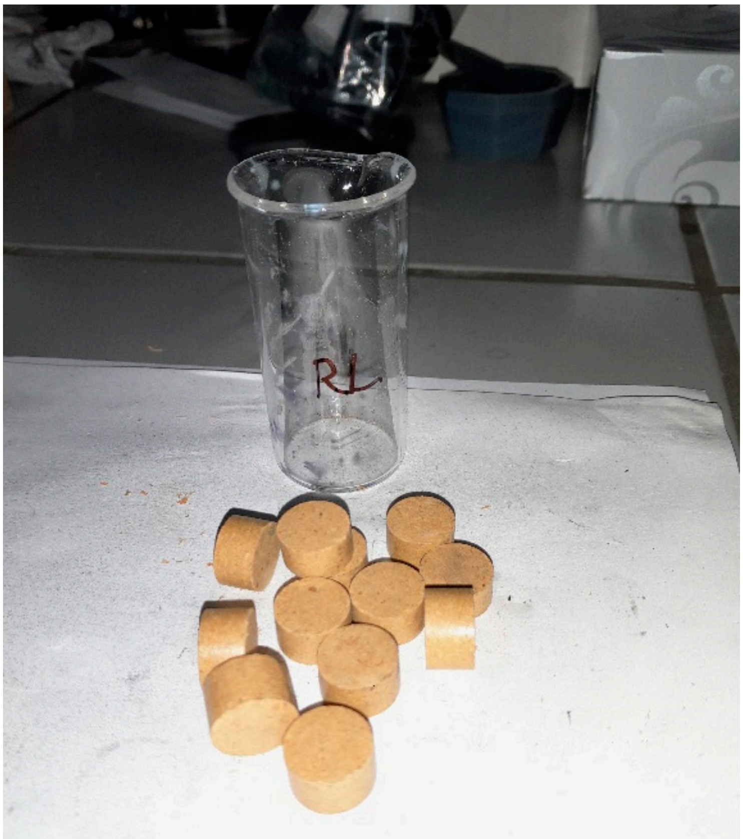

Molded knotweed leaves

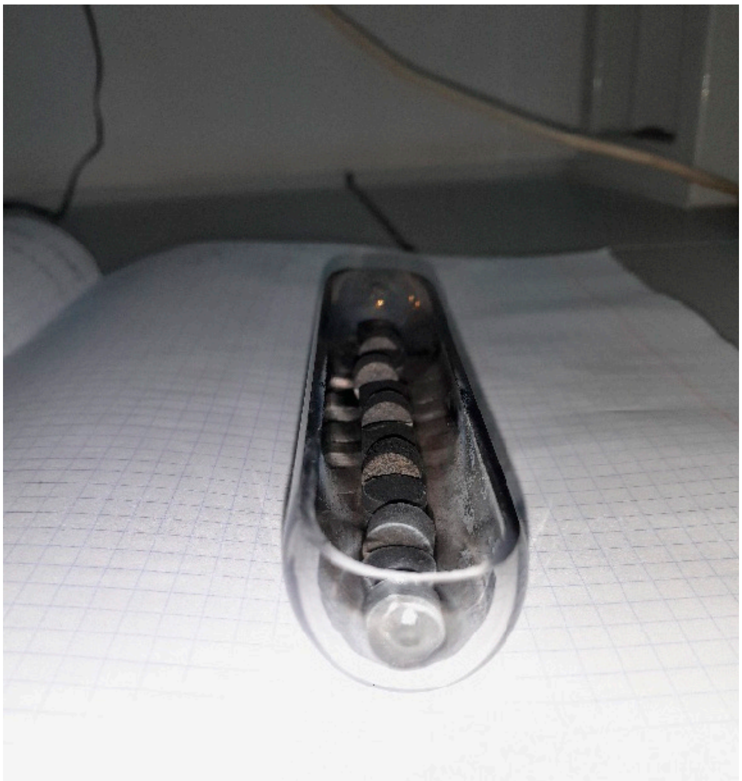

Pyrolyzed knotweed leaves

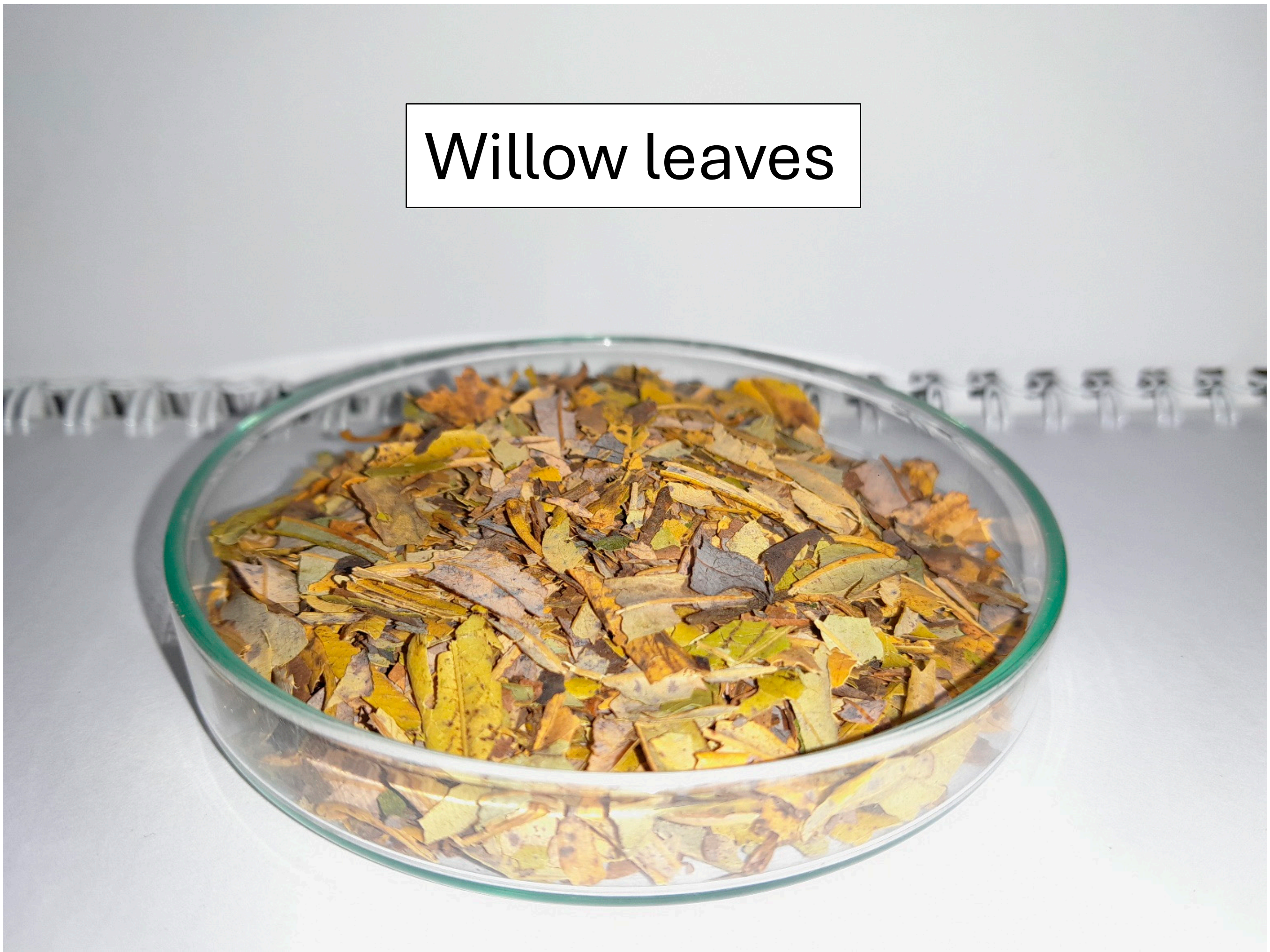
Willow leaves

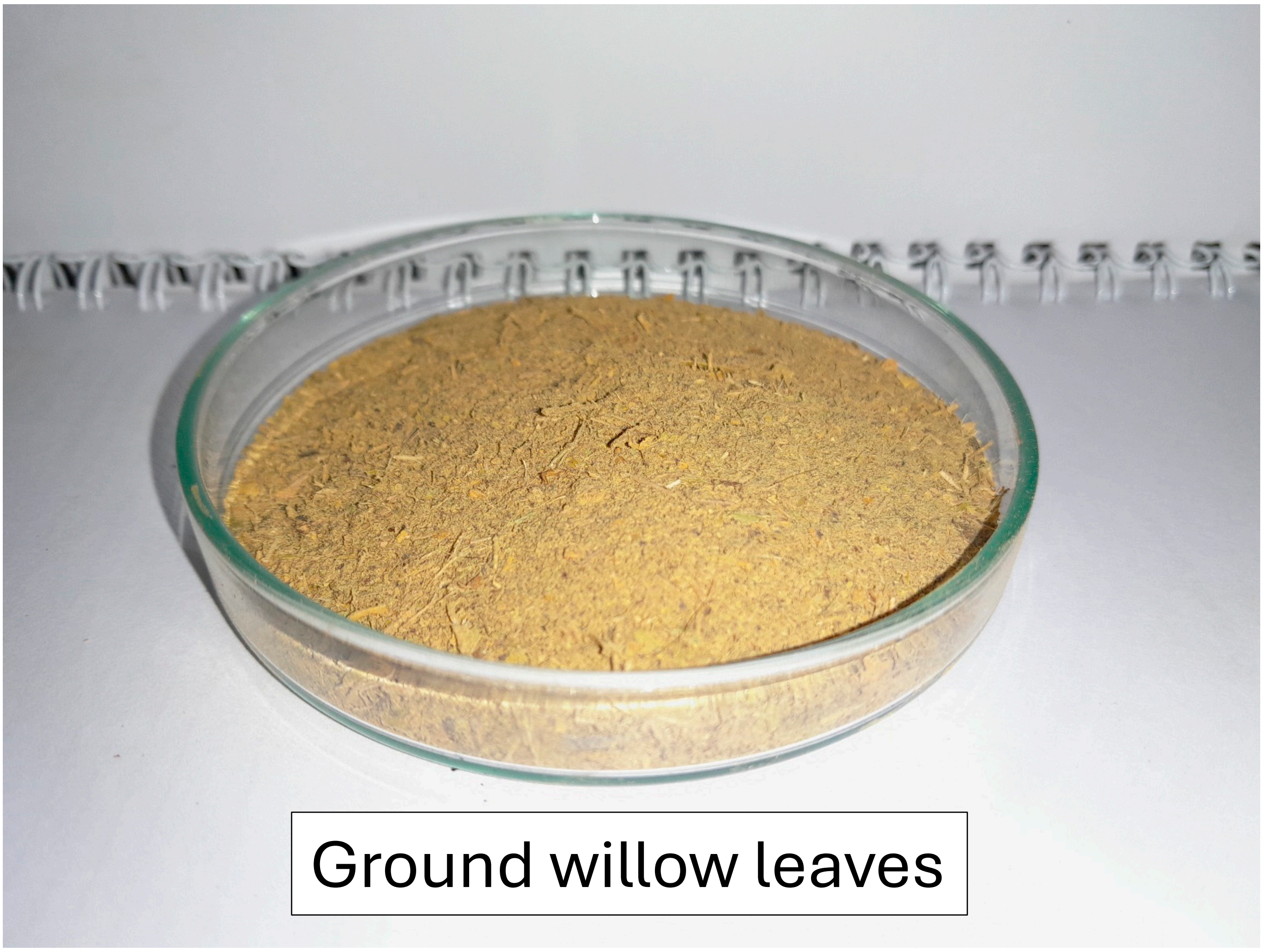
Ground willow leaves

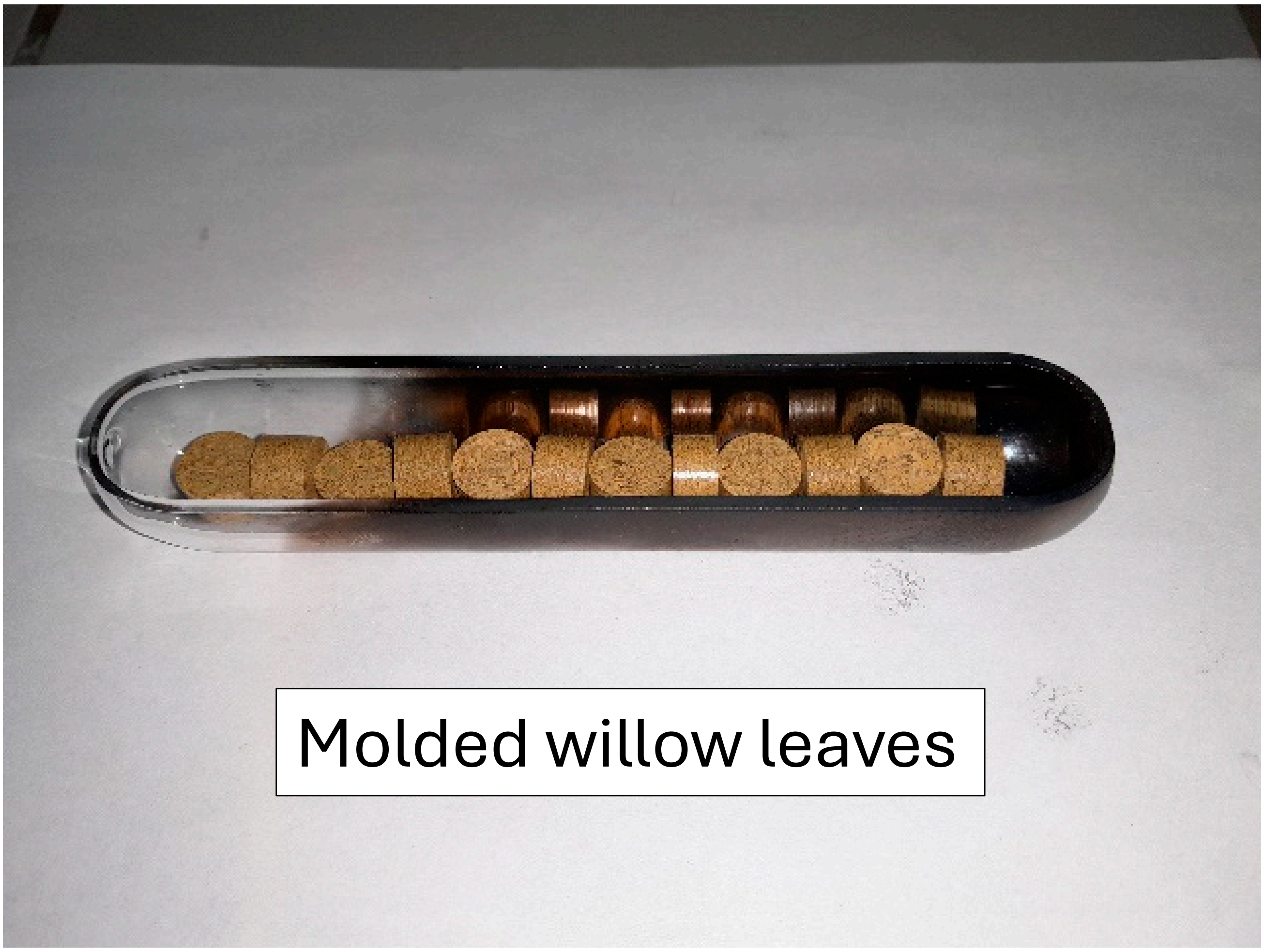
Molded willow leaves

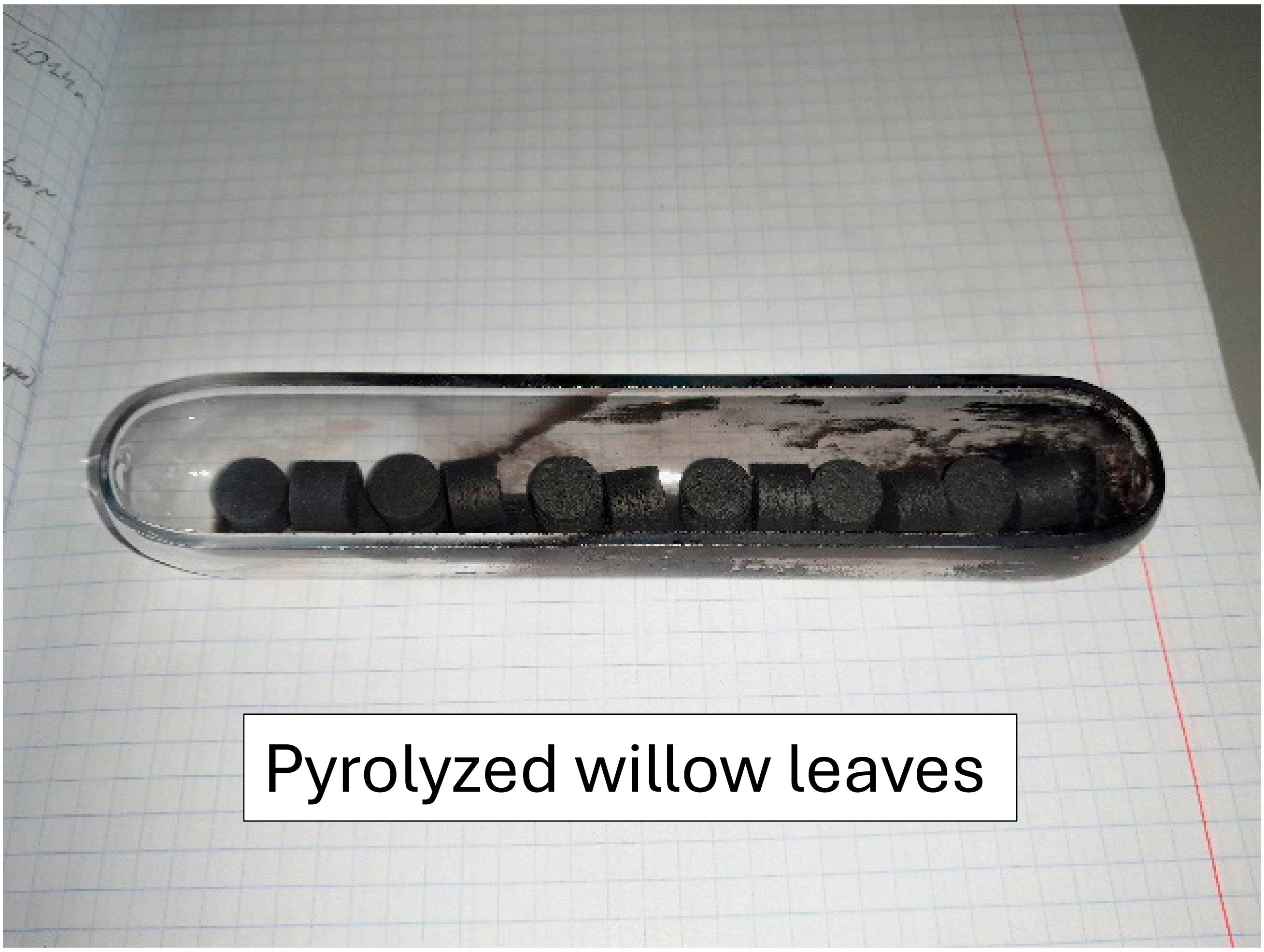
Pyrolyzed willow leaves

Pyrolysis table

Material	Mass before pyrolysis [g]	Mass after pyrolysis [g]	Mass reduction [%]
Maple leaves (KL)	14.9825	4.6474	69
Knotweed leaves (RL)	13.7561	4.2604	69
Willow leaves (WL)	16.0704	4.8317	70

XRD tables

1.

Nickel-impregnated carbons				
**Sample name**	**Peak number**	**2 Theta Angle [°]**	**Crystallographic phase**	**Card number**
Ni@ML	1	36.85	Nickel carbide (1,0,4)	01-072-1467
	2	42.97	Nickel carbide (0,0,6)	01-072-1467
	3	46.96	Nickel carbide (2,0,2)	01-072-1467
	4	62.50	Nickel carbide (0,1,8)	01-072-1467
	5	75.44	Nickel carbide (2,0,8)	01-072-1467
	1’	28.90	Calcium oxide	00-021-0155
	2’	46.96	Calcium oxide	00-021-0155
	3’	62.50	Calcium oxide	00-021-0155
	4’	75.44	Calcium oxide	00-021-0155
Ni@KL	1	22.80	Nickel carbide (0,1,2)	01-072-1467
	2	28.98	Nickel carbide (1,1,1)	00-045-0979
	3	35.69	Nickel carbide (1,1,4)	00-045-0979
	4	39.05	Nickel carbide (2,0,4), (1,1,0)	00-045-0979, 01-072-1467
	5	42.88	Nickel carbide (2,0,5), (0,0,6)	00-045-0979, 01-072-1467
	6	46.95	Nickel carbide (1,2,3), (2,0,2)	00-045-0979, 01-072-1467
	7	60.65	Nickel carbide (0,1,8)	01-072-1467
	8	94.70	Nickel carbide (1,3,4)	01-072-1467

Ni@WL	1	29.65	Nickel carbide (1,1,1)	00-045-0979
	2	36.16	Nickel carbide (1,1,4)	00-045-0979
	3	39.62	Nickel carbide (2,0,4)	00-045-0979
	4	43.39	Nickel carbide (2,0,5)	00-045-0979
	5	44.74	Nickel carbide (1,1,1)	01-074-5561
	6	47.57	Nickel carbide (0,0,9)	00-045-0979
	7	52.04	Nickel carbide (2,0,0)	01-074-5561
	8	76.59	Nickel carbide (2,2,0)	01-074-5561
	9	93.09	Nickel carbide (3,1,1)	01-074-5561
	1’	37.27	Nickel oxide (1,1,0)	01-072-1464
	2’	43.39	Nickel oxide (1,1,-1)	01-072-1464
	3’	63.02	Nickel oxide (0,2,0)	01-072-1464

2.

**Non-impregnated carbons**				
**Sample name**	**Peak number**	**2 Theta Angle [°]**	**Crystallographic phase**	**Card number**
ML	A	24.95	graphite	01-074-2329
	B	27.08	Carbon(0,0,4)	00-026-1080
	C	32.68	Lignite(NR)	00-005-0625
	D	43.96	Graphite	00-003-0401
	E	49.98	carbolite(5,1,1)	00-050-0927
	F	55.73	Carbon(1,0,5)	00-026-1080
	G	73.65	carbolite(4,5,2)	00-050-0927
	H	84.75	Carbon(1,1,4)	00-026-1080
	I	87.79	Carbon	00-005-0625

KL	A	18.47	Carbon(1,0,4),Carbon (20)(1,0,0)	00-048-1449,01-081-8955
	B	25.25	Carbon Nanotubes(0,0,2),Carbon(1,0,7), Carbon (20)(1,1,0)	00-058-1638,00-048-1449,01-081-8955
	C	27.96	Carbon(2,0,5),Carbon (20)(1,1,1)	00-048-1449,01-081-8955
	D	33.14	Carbon	00-046-0943
	E	34.46	Carbon (20)(2,0,0)	01-081-8955
	F	40.98	Carbon	00-026-1078
	G	44.08	Carbon Nanotubes(1,0,0),Carbon (20)(2,1,1)	01-071-3649,00-058-1638,01-081-8955
	H	47.53	Carbon	00-020-0258
	I	50.43	Carbon (20)(2,2,0)	01-081-8955
	J	62.66	Carbon (20)(2,2,2)	01-081-8955
WL	A	19.18	buckminsterfullerene(2,2,0)	00-044-0558
	B	27.38	buckminsterfullerene(3,3,1)	00-044-0558
	C	33.63	buckminsterfullerene(4,2,2)	00-044-0558
	D	43.74	Carbon(1,1,1),buckminsterfullerene(5,3,3)	00-060-0053,00-044-0558
	E	50.40	Carbon(2,0,0),buckminsterfullerene(6,4,0)	00-060-0053,00-044-0558
	F	73.89	Carbon(2,2,0),buckminsterfullerene(10,4,0)	00-060-0053,00-044-0558
